# Identification of Photochemically Generated Volatile Species of Ruthenium and Osmium Using Direct Analysis in Real Time Mass Spectrometry

**DOI:** 10.1021/acs.analchem.5c02929

**Published:** 2025-07-28

**Authors:** Ignacio Machado, Beatrice Campanella, Zhendong Lyu, Stanislav Musil

**Affiliations:** a Institute of Analytical Chemistry of the Czech Academy of Sciences, Veveří 97, Brno 602 00, Czech Republic; b Faculty of Chemistry, Universidad de la República, Analytical Chemistry Area, Gral. Flores 2124, Montevideo 11800, Uruguay; c Institute of Chemistry of Organometallic Compounds, 9327 National Research Council, via Giuseppe Moruzzi 13, Pisa 56124, Italy; d Department of Analytical Chemistry, Charles University, Faculty of Science, Hlavova 8, Prague 128 43, Czech Republic

## Abstract

Photochemical vapor generation (PVG) was coupled to direct analysis in real time (DART) high-resolution mass spectrometry (HRMS) using N_2_ as the discharge gas in an attempt to identify unknown volatile carbonyls of Ru and Os generated during the UV photolysis of HCOOH-based photochemical media previously described in the literature. Initial insights into the ambient ionization process in the N_2_ DART were gained using volatile W­(CO)_6_ and Fe­(CO)_5_, either photochemically generated or introduced as standards from a headspace. In general, significant changes in the original carbonyl structure are observed in both positive and negative ion modes, characterized by the loss of CO group(s), oxidation, hydration, and formation of various adducts derived from N_2_ used as the discharge gas. Nevertheless, the ions detected under PVG conditions based on dilute or concentrated HCOOH media, preferably in the presence of transition metal mediators, suggest that the generated carbonyls of Ru and Os are mononuclear, contain five carbonyl groups, and are therefore Ru­(CO)_5_ and Os­(CO)_5_. When Co^2+^ was used as a mediator, some difficulties in identification were encountered because volatile Co­(CO)_4_H was cogenerated with significant efficiency, overloading the DART and HRMS and even resulting in mixed metal carbonyl cluster ions during ionization. Additional experiments with PVG of Os conducted under oxidative conditions using deionized water, dilute HNO_3_, and dilute H_2_O_2_ as the photochemical media confirmed OsO_4_ as the volatile species. The same volatile species was also identified as the dominant product using dilute CH_3_COOH with the addition of Fe^2+^ as a mediator, suggesting the rather oxidative nature of this medium, although some distinct carbonyl/hydrido/methyl or acetato species were also observed. The controversies are discussed as well as other peculiarities of the DART-HRMS technique for the identification of volatile metal carbonyls.

## Graphical abstract

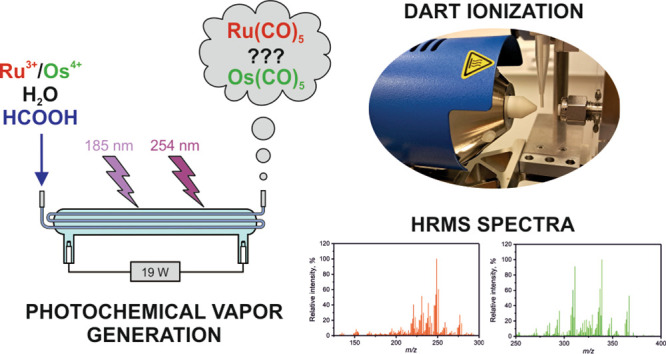

## Introduction

Photochemical vapor generation (PVG) is an alternative sample introduction technique for analytical atomic spectrometry that provides an enhanced performance by increasing analyte introduction efficiency and removing liquid matrix components that can otherwise interfere with their accurate detection. It is now applicable to a broad range of elements (approximately 30), including hydride-forming elements, nonmetals, and many transition metals.^1^ The analyte is converted to volatile species during UV irradiation of the liquid photochemical medium containing low molecular weight organic acid, such as HCOOH. The UV photolysis of undissociated HCOOH and dissociated HCOO^–^ yields strongly reducing radical species (H^•^ and CO_2_^•–^) and aquated electrons (e_(aq)_^–^), according to reactions 1–5:^1,2^

HCOOH→hνCHO•+OH• 1

$$\mathrm{HCOOH}\overset{h\nu }{\to }\mathrm{HCO}O^{•}+H^{•}$$ 2

HCOOH→hνCOOH•+H• 3

$$\mathrm{HCO}O^{-}\overset{h\nu }{\to }{\mathrm{CO}}_{2}^{•-}+H^{•}$$ 4

$$\mathrm{HCO}O^{-}\overset{h\nu }{\to }\mathrm{HCO}O^{•}+e_{\left(aq\right)}^{-}$$ 5

which reduce the ionic analytes (M^n+^) to the elemental state (M^0^). In addition, gases (H_2_, CO, and CO_2_) are produced in the liquid media,^3^ as the end products of radical reactions 6–14:

$$H^{•}+H^{•}\to H_{2}$$ 6

$$\mathrm{HCO}O^{-}+H^{•}\to CO_{2}^{•-}+H_{2}$$ 7

CHO•→H•+CO 8

COOH•→OH•+CO 9

CHO•+CHO•→HCHO+CO 10

CHO•+COOH•→HCOOH+CO 11

$$\mathrm{HCO}O^{•}+H^{•}\to H_{2}+CO_{2}$$ 12

$$\mathrm{HCO}O^{•}+\mathrm{HCO}O^{•}\to \mathrm{HCOOH}+CO_{2}$$ 13

$$\mathrm{HCO}O^{•}+CO_{2}^{•-}\to \mathrm{HCO}O^{-}+CO_{2}$$ 14

Some of them may be involved in the formation of resulting volatile species. If a higher order carboxylic acid, such as CH_3_COOH, is used, then ^•^CH_3_ must also be considered, possibly resulting in CH_4_ and C_2_H_6_ as gaseous products.^3^ Depending on the element (analyte) and photochemical medium used, the following volatile species have been generated: free atoms of Hg, hydrides or alkylated species ofhydride-forming elements and nonmetals, and carbonyls oftransition metals and Se.^1^ Oxidative conditions have also been advantageously used for the PVG of volatile OsO_4_ from dilute H_2_O_2_, HNO_3_, or pure water.^4–6^

The inherent advantages of the PVG technique for ultrasensitive determination and even speciation analysis have been well demonstrated in recent years,^7–12^ but parallel attempts focused to better understand the PVG reaction mechanism have always lagged behind.^3,13–21^ Despite the recent success in detecting enhanced production of reducing radicals (e.g., CO_2_^•–^) using an electron paramagnetic resonance spin trapping technique,^13,14,16–18,21–23^ there remains a significant gap in the explanation of the beneficial effects of transition metals as mediators.

To thoroughly understand the individual PVG reaction pathways and the roles of radicals, e_(aq)_^–^, and generated gases, it is crucial to know the identity of the final volatile products. The majority of the volatile species generated by PVG have been convincingly identified using gas chromatography mass spectrometry (GC-MS), including As, Bi, Br, Co, I, F, Fe, Mo, Ni, Os (as OsO_4_), Sb, Se, Te, and W using either a direct GC-MS sampling of the effluent gases from the photoreactor or following their cryotrapping.^14,16,22,24–36^ (Note: Only the most important works are referenced). However, this approach was not successful in identifying the volatile species of Ru^9,23,33^ and Os^4,23,33^ generated under reductive PVG conditions resulting from the presence of HCOOH in the photochemical media, despite their high PVG efficiencies. Based on the reactions 6–14, it can be assumed that UV photolysis of HCOOH-based media can only produce hydrided/carbonylated Ru and Os compounds with the general formula M_*x*_(CO)_*y*_H_*z*_, assuming that the 18-valence electron rule is satisfied and carbon dioxide remains inert and does not bind to the metal. The whole process of PVG can be described by the simplified scheme 15:

$$M^{n+}\overset{e_{\left(aq\right)}^{-}/CO_{2}^{•-}/H^{•}}{\to }M^{0}\overset{+CO/H_{2}}{\to }M_{x}{\left(\mathrm{CO}\right)}_{y}H_{z}$$ 15,

where n = 3 or 4 for Ru and n = 4 for Os. The simplest possibilities are the mononuclear Ru­(CO)_5_ and Os­(CO)_5_, described as liquids (melting points –16 to –17 °C and 2 to 2.5 °C, respectively) but with high vapor pressures, and although somewhat elusive, sufficiently characterized in older papers.^37–41^ Other options are stable trinuclear Ru_3_(CO)_12_ and Os_3_(CO)_12_ utilized as catalysts in organic synthesis. Since UV photolysis of HCOOH produces a significant amount of H_2_ in addition to CO (reactions 6–12),^3^ incorporation of H_2_ into the metal carbonyl structure instead of CO may occur.^37,42,43^ In the case of Os, when its volatile species is generated from CH_3_COOH medium,^4^ the CH_3_ group should also be considered.

The reason for the unsuccessful GC-MS identification of Ru and Os carbonyl species may be due to their low (thermal) stability, incompatible with typical temperature-controlled GC programs, or rapid decomposition on the surfaces of the GC apparatus. Alternative techniques, such as UV–vis spectrometry and high-resolution mass spectrometry with electrospray ionization (ESI-HRMS), were employed by Yang et al.^23^ They cryogenically trapped volatile species of Ru in acetonitrile and because metal carbonyls are nonpolar and thus directly undetectable by ESI-MS, Ag^+^ was added to promote ionization, according to a previously reported protocol.^44,45^ The ESI-HRMS spectra indicated the presence of polynuclear Ru species, most likely Ru_3_(CO)_12_, but the authors themselves noted that the identification was not entirely conclusive. Therefore, they only hypothesized the presence of some Ru_*x*_(CO)_*y*_ species.^23^ The weakness of this derivatization approach based on Ag^+^ addition is that it is not a general technique. It is mainly suitable for ESI ionization of polynuclear metal carbonyls but not of simple mononuclear carbonyls, such as W­(CO)_6_.^44,45^ Due to the instability of Ru­(CO)_5_, there is also a high risk of clustering to Ru_3_(CO)_12_ after trapping it in the liquid acetonitrile. Additionally, Yang et al.^23^ examined the effluents from the photoreactor for the presence of OsO_4_ in the various photochemical media. The evidence was based on the oxidation of added quercetin to quinone species by generated OsO_4_ and the appearance of a characteristic UV absorption band at 291 nm. This band was clearly observed when PVG was conducted from DIW and surprisingly also from dilute CH_3_COOH medium in the presence of Fe^3+^ as the mediator, indicating the oxidative nature of these conditions.

Clearly, there is an urgent need for a more technologically capable approach to effectively identify less stable volatile species of metals. It is also imperative to clarify the remaining gaps regarding the identities of volatile species of Ru and Os generated from various media.^4,9,15,23^ In this work, the identification of volatile species of Ru and Os was attempted using an ambient ionization technique - direct analysis in real time coupled to high-resolution mass spectrometry (DART-HRMS). DART is an atmospheric pressure ionization source that has become an established technique for the MS analysis of typically organic solid, liquid, and gaseous samples due to its simplicity and speed.^46^ Electronically excited (metastable) species are produced in a corona discharge operated in He or N_2_, typically preheated to 50–550 °C,^46,47^ wherein the DART source may generate positive or negative ions depending on the applied potential. In the positive ion mode, the excited-state He atoms (He*) induce Penning ionization of ambient atmospheric water molecules, generating protonated water clusters, followed by proton transfer to the analyte. Nitrogen as a discharge gas has been much less studied in DART-MS.^48^ It has a number of long-lived vibronic excited states and the maximum ionization energy available for Penning ionization by metastable N_2_* is given as 11.5 eV, much lower than that of He* (19.8 eV) and lower than that required to ionize water (12.6 eV). However, H_3_O^+^ can be detected in the background mass spectrum with N_2_ as the discharge gas. It possibly results from multiple pathways involving Penning ionization of N_2_, dimerization of N_2_, and subsequent ionization of water.^46^ Ultimately, this leads to the formation of H_3_O^+^ and other protonated water clusters that enable proton transfer to the analyte. In addition, concurrent ionization realized by charge transfer from generated N_4_^+•^, O_2_^+•^, or NO^+^ can be observed. In the negative ion mode, the generation of negatively charged ions proceeds via electron capture, dissociative electron capture, deprotonation transfer, and anion attachment. Atmospheric O_2_ is the preferred species to undergo the initial electron capture to form O_2_^–•^ that can make adducts with the analyte.^47,49^ In addition to O_2_^–•^, the background ions generated from atmospheric components include NO_2_^–•^, NO_3_^–•^, and others, depending on traces of solvents. All of them may also form adducts with the analyte.^46^

DART-MS has been used less frequently to detect or identify gaseous inorganic or organometallic compounds. Borges et al. reported DART-MS spectra of several organometallic compounds of As, Fe, Hg, Pb, Se, and Sn and systematically studied the influence of the operating parameters of the DART source.^50^ Vyhnanovský et al. successfully used DART-HRMS to identify the volatile Pd chelate species after chemical vapor generation (CVG) of Pd by tetrahydridoborate using diethyldithiocarbamate as a modifier.^51^ However, for simple gaseous hydrides, such as SeH_2_, BiH_3_, and AsH_3_ (or its methylated analogues), numerous ion species were shown to originate in the DART,^52–56^ with pronounced oxidation, hydrogen abstraction, loss of methyl group(s), and formation of oligomeric ions complicating identification of the original species.

## Experimental Section

### Chemicals

Analytical standards, chemicals used to prepare photochemical media, and compounds used as metal ion mediators are detailed in the Supporting Information.

### Photochemical Vapor Generator

The generator in a flow injection (FI) mode of operation (0.5 mL sample volume) was based on the system used in our previous studies^8,9,11,15^ and is illustrated in Figure S1. It comprised a thin-film flow-through photoreactor (Jitian Instruments Co., China) and two gas-liquid separators (GLS I and II) in series. The GLS I was cooled in an ice-water bath throughout the experiment. The GLS II allowed the simultaneous introduction of the headspace samples of solid or liquid metal carbonyl standards through a GC septum using a Hamilton syringe. The photochemical medium was delivered to the photoreactor at 2 mL min^–1^ (corresponding to an irradiation time of ≈22 s) using a peristaltic pump. Details of the generator and operating parameters are given in the Supporting Information.

### Direct Analysis in Real Time Mass Spectrometry

A DART ion source (model SVPS-300, Ionsense, USA) equipped with a Vapur Interface was connected to an Orbitrap Exploris 120 high-resolution mass spectrometer (HRMS, Thermo Fisher Scientific, Inc.) to identify the generated volatile species. The measurements were conducted using two configurations of the DART when coupled to PVG, i.e., either a standard “open” environment for ionization (Figure 1) as described earlier,^51,54^ or a “confined” setup (Figure S2) similar to the “tee-shaped” device employed recently for analysis of complex gaseous samples.^57^ The details on these configurations and the Vapur Interface are given in the Supporting Information.

**Figure 1:**
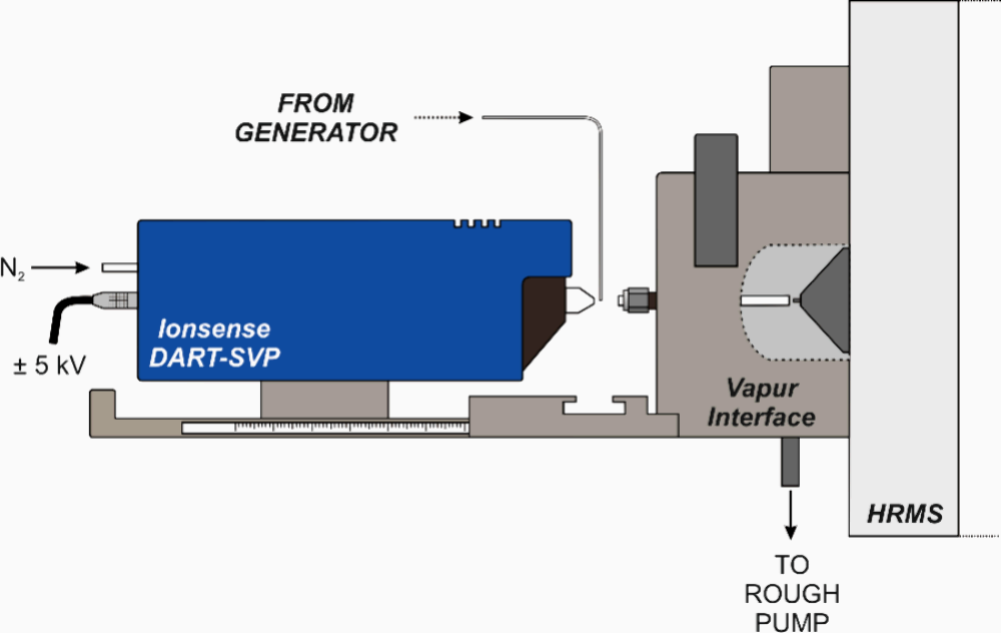
Open setup of DART for coupling to FI-PVG. The layout of the interior of the Vapur Interface is displayed for clarity in the window defined by the dashed line.

The potential applied to the DART needle electrode to sustain the corona discharge was positive or negative 3.5 kV, depending on the ion mode used. The voltages on a grid electrode positioned downstream of the plasma discharge were typically set to +350 V or –350 V unless explicitly stated otherwise. Nitrogen (99.9990% purity, SIAD Ltd., Czech Republic) was used as the discharge gas at approximately 2.5 L min^–1^ and heated to arbitrary temperatures. In some experiments, N_2_ was substituted for Ar of the same purity. (Note: Helium could not be used as a discharge gas in combination with the Orbitrap Exploris 120 HRMS because He gas causes a false reading of a UHV pressure sensor and measurement is not enabled).

Full scan mass spectra were acquired at a resolution of 120000 (at 200 *m*/*z*). Tuning of the Orbitrap Exploris 120 HRMS and typical settings are described in the Supporting Information.

### Measurement Procedure and Evaluation

The photochemical medium was continuously pumped into the photoreactor and the analyte standard, prepared in the photochemical medium and possibly spiked with selected metal mediators, was manually injected into the carrier stream at the beginning of a PVG cycle and recording of the DART-HRMS spectra. Recording was stopped after all transient signal intensities returned to the baseline. The elemental composition of ions formed in the DART from individual volatile species was identified by accurate mass measurements and consistent deviation from the theoretical ion mass (Δm shown in ppm units in Tables). Typically, only the isotopes of the analytes (and mediators), H, C, N, and O were considered for that purpose, i.e., elements that may be derived from the analyte/mediator, photochemical media used for PVG, discharge gas, and air. The observation of characteristic FI peak profiles in any *m*/*z* window facilitated identification of the generation process, attributed to the volatile analyte species coming from the generator, due to the added dimension of time discrimination. Furthermore, the investigated analytes exhibit characteristic isotopic patterns, ensuring unequivocal identification of prospective volatile species, namely 5 isotopes for W: ^184^W (natural abundance 30.6%), ^186^W (28.4%), ^182^W (26.5%), ^183^W (14.3%), and ^180^W (0.12%); 4 isotopes for Fe: ^56^Fe (91.8%), ^54^Fe (5.85%), ^57^Fe (2.12%), and ^58^Fe (0.28%); 7 isotopes for Ru: ^102^Ru (31.6%), ^104^Ru (18.6%), ^101^Ru (17.1%), ^99^Ru (12.8%), ^100^Ru (12.6%), ^96^Ru (5.5%) and ^98^Ru (1.9%); and 7 isotopes for Os: ^192^Os (40.8%), ^190^Os (26.3%), ^189^Os (16.2%), ^188^Os (13.2%), ^187^Os (2.0%), ^186^Os (1.6%), and ^184^Os (0.02%). The full mass spectra were evaluated in the Qual Browser software in the time range of the maximum signal intensities of the ions (i.e., at the maximum of the FI peaks) and corrected for the averaged background spectra taken before and after the peak appearance. Due to the large number of ions generated from volatile analyte species in the DART, only the ions corresponding to the most abundant analyte isotopes, i.e., ^184^W, ^56^Fe, ^102^Ru, ^192^Os, with relative intensities greater than 5% are listed in the Tables.

A DART-HRMS interrogation of solid W­(CO)_6_, Ru_3_(CO)_12_, and Os_3_(CO)_12_ or liquid Fe­(CO)_5_ standards was conducted by Hamilton syringe sampling of 5–150 μL of headspace above 1–10 mg of solid or liquid metal carbonyls placed in 2 mL vials and immediately introducing it either through the GC septum into the GLS II, or directly to the N_2_ stream of the DART (open setup), while PVG from blank (photochemical medium) was still in operation. Unless otherwise stated, the vials with solid Ru_3_(CO)_12_ and Os_3_(CO)_12_ were heated to about 125 °C.

## Results and Discussion

### Effects of Configuration and Parameters on DART-HRMS Spectra

The standard open setup of the DART was used almost exclusively for measurements because it also allowed the headspace of metal carbonyl standards to be introduced into the DART for comparison of the spectra. The position of the PTFE tube tip relative to the angled ceramic cap of the DART source and the edge of the ceramic capillary of the Vapur Interface (see Figure 1) was optimized for maximum intensity of ions. The confined setup was only used in some experiments to provide supporting data on the ionization processes. In general, the measurements with the confined setup were characterized by several times higher intensities of analyte ions (absolute signal intensities are given in the footnotes of the Tables), but also by an order of magnitude higher intensities of background ions and thus significantly higher noise.

The parameters that affect the DART ionization of volatile species and ion transfer for the HRMS detection were examined for each analyte and PVG conditions studied. Results are typically presented for conditions chosen as a compromise between the sensitivity of the most abundant ion and minimal changes in “structural” information reflected in the loss of one or more CO (carbonyl) groups and their substitution by, for example, hydration and/or oxidation. The main impacts arose for the discharge gas and transfer tube temperatures, the selected values of which thus slightly vary among the analytes and PVG conditions. The effect of other parameters was minimal (e.g., the potential applied to the grid electrode of the DART source), or the observed spectra followed the similar trend of severe fragmentation (cone voltage and RF lens voltage), and such general observations are detailed in the Supporting Information. In addition, the concentration of an analyte taken for PVG was optimized to minimize the formation of dimeric and trimeric species by aggregation of ions with neutral species, to less than 5% of the relative intensity of the monomer species.

### PVG Conditions Studied and Preliminary Experiments

Based on recent studies,^4,9,15,23^ the gas phase resulting from PVG using different photochemical media was interrogated with DART-HRMS in an attempt to identify the generated volatile species of Ru and Os. The main focus was on reductive PVG conditions arising from the use of HCOOH in the media. The photochemical media studied for Ru included 8 M HCOOH with the addition of 10 mg L^–1^ Co^2+^ and 25 mg L^–1^ Cd^2+^ as mediators to the sample,^9^ hereafter expressed in the text as 8 M HCOOH (10Co/25Cd); then 0.01 M HCOOH (10Cd)^15^ and 0.005 M HCOONa.^15^ For Os, the main focus was placed on the reductive PVG conditions recently described by Yang et al. and de Oliveira et al.: 30% (v/v) HCOOH (50Co/20Cd),^23^ 1% (v/v) CH_3_COOH (50Fe),^4^ and 2% (v/v) HCOOH.^4^ In some cases, it was necessary to adjust the composition (or concentration) of the added mediators, which is justified in the text. Although the volatile OsO_4_ generated under oxidative PVG conditions, using 5% (v/v) HNO_3_,^4,5^ was recently convincingly identified by GC-MS,^33^ DIW and 1% (v/v) H_2_O_2_ were also examined^4,6^ and the results were compared to those obtained with 5% (v/v) HNO_3_.

Significantly higher concentrations (typically 0.1–5 mg L^–1^) of all analytes had to be used with DART-HRMS due to its lower sensitivity compared to previous ICPMS studies.^4,9,15,23^ A concentration of 5 mg L^–1^ was used as the upper limit, because at higher concentrations a downward curvature or even rollover of the calibration function constructed from the maxima of the ion intensities of the measured transient signals was observed, probably due to pronounced aggregation of reduced metals in the liquid photochemical medium. Despite the higher analyte concentrations, the PVG efficiencies for Ru obtained under various reductive conditions were possibly in the range 8–25% (see Table 1 in ref 15). Since the PVG of Os has not been optimized in our laboratory yet, we only estimated the PVG efficiencies for the above media and sample flow rate of 2 mL min^–1^ using the approach based on a comparison of the sensitivities obtained with PVG and pneumatic nebulization (PN) both simultaneously coupled to ICPMS.^8,9,11,58^ Details of the coupling of FI-PVG to ICPMS and the measurement parameters are described in the Supporting Information and Table S1. The following PVG efficiencies were determined: ≈2% for DIW, ≈6% for 5% (v/v) HNO_3_, ≈2.6% for 1% (v/v) CH_3_COOH (50Fe), ≈26% for 30% (v/v) HCOOH (50Co/20Cd), and <1% for 2% (v/v) HCOOH. These values were sufficient to enable measurements with DART-HRMS.

Due to limited experience with ionization of volatile metal carbonyl species in the DART, W­(CO)_6_ and Fe­(CO)_5_ were used as the model volatile species because they had previously been identified by GC-MS during PVG of W and Fe.^31,33^ The details of the experiments are given in the Supporting Information, Figures S3A,B and S4, and Tables S2, S3, and S4. In brief, DART ionization induced significant structural changes in these volatile metal carbonyls, which can be characterized by severe fragmentation, oxidation, hydration, and formation of various adducts derived from N_2_ used as the discharge gas. Despite these changes, however, it appears feasible to use DART-HRMS to identify mononuclear volatile metal carbonyls, as the number of CO groups bound to the central atom can be deduced from certain ions detected.

### Ruthenium

The first PVG conditions that were investigated for Ru were 0.01 M HCOOH (10Cd). These conditions provided clear DART-HRMS spectra due to the low concentration of HCOOH used and no volatile species cogenerated from added Cd^2+^. Positive and negative ion mode spectra were acquired using 1 and 0.2 mg L^–1^ Ru for PVG, respectively, and are presented in Figure 2A,B, with the most intense ions being summarized in Tables S5 and S6.

**Figure 2:**
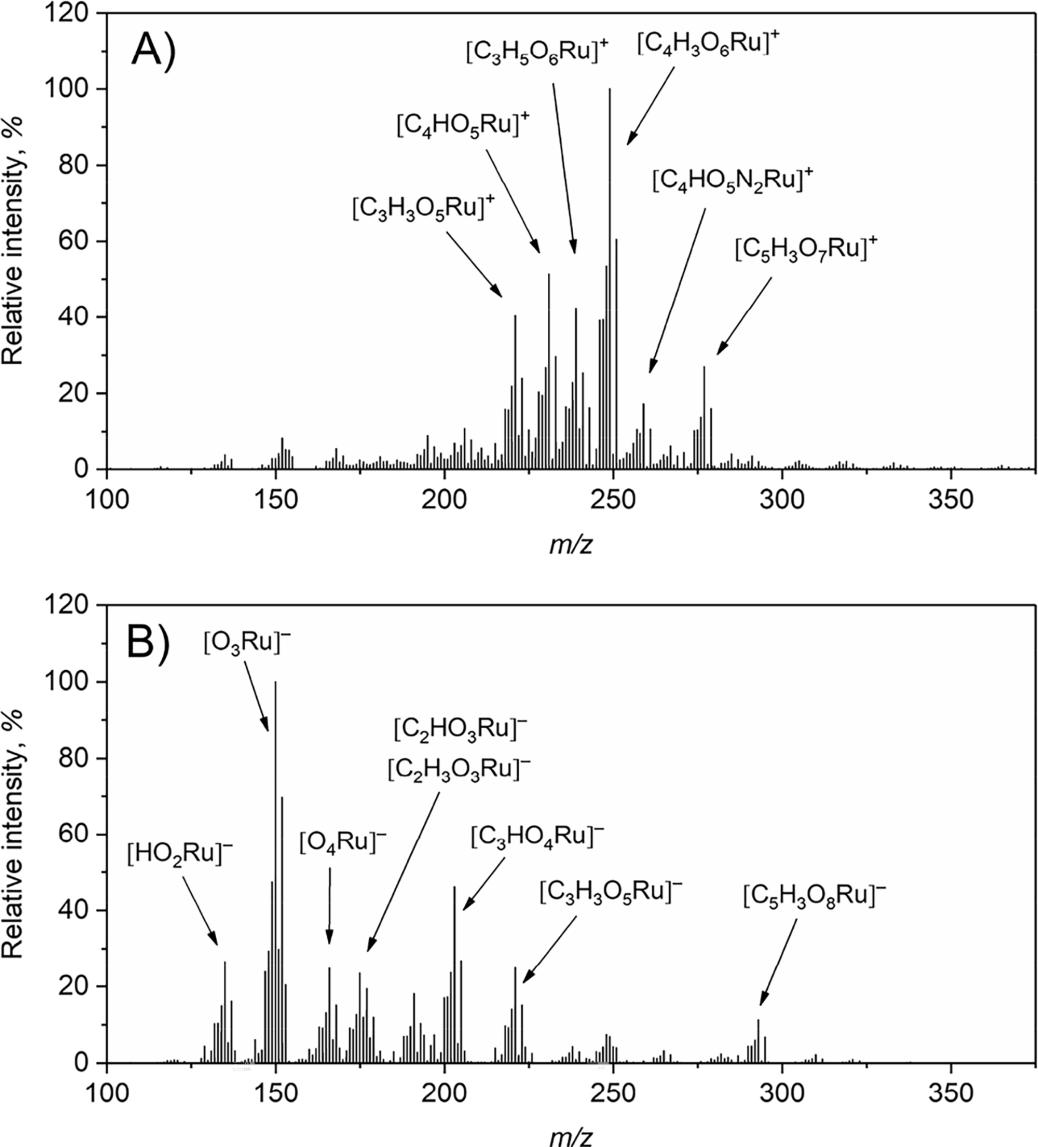
Full scan DART-HRMS spectra obtained in the A) positive and B) negative ion mode during FI-PVG of Ru using 0.01 M HCOOH (10Cd) and the open setup of the DART. Conditions: 1 and 0.2 mg L^–1^ Ru taken for PVG in the positive and negative ion mode, respectively, sample flow rate 2 mL min^–1^; N_2_ discharge gas temperature 150 °C, ion transfer tube temperature 200 °C. Only some ions are labeled, see Tables S5 and S6 for others.

The most abundant species (100% relative abundance) in the positive ion mode was [C_4_H_3_O_6_Ru]^+^, while [O_3_Ru]^−^ dominated in the negative ion mode. In both modes, the ions [C_5_H_3_O_7_Ru]^+^ and [C_5_H_3_O_8_Ru]^−^ containing 5 carbon atoms, and thus likely 5 CO groups, were highly abundant (27% and 11%, respectively). These were also the ones with the highest *m*/*z* detected with significant relative intensities. It appears that the ion [C_5_H_3_O_7_Ru]^+^ corresponds to [Ru­(CO)_5_+H_2_O+OH]^+^. For some ions, maintaining 3 and 4 carbons, the trend of addition of a single H_2_O molecule is evident, leading to [Ru­(CO)_*x*_+y­(H_2_O)+OH]^+^, where x = 3 and 4 and y = 0, 1, 2, and 3, e.g., [C_3_HO_4_Ru]^+^ (6.7%), [C_3_H_3_O_5_Ru]^+^ (40%), [C_3_H_5_O_6_Ru]^+^ (42%), and [C_3_H_7_O_7_Ru]^+^ (11%). In the negative ion mode (Figure 2B and Table S6), more significant fragmentation and oxidation occurred than in the positive ion mode, probably due to the action of generated O_2_^–^^•^. For some ions that still retain several CO groups, OH^–^ adducts are evident in the spectrum. For example, the intense ions [C_3_HO_4_Ru]^−^ (46%) and [C_3_H_3_O_5_Ru]^−^ (25%) may correspond to [Ru­(CO)_3_+OH]^−^ and [Ru­(CO)_3_+H_2_O+OH]^−^, respectively, while the ions [C_5_H_3_O_8_Ru]^−^ may correspond to [Ru­(CO)_5_+H_2_O+O+OH]^−^. Interestingly, quite many N and even N_2_ containing ions were detected in the positive ion mode. Some of them (e.g., [H_4_O_3_NRu]^+^ (5.3%)) may be NH_4_^+^ adducts, while the other ions (e.g., [C_2_H_2_O_4_NRu]^+^ (11%) and [C_4_HO_5_N_2_Ru]^+^ (17%)) can be formed by substitution of CO by NO^+^ and N_2_ from the discharge gas as these ligands are isoelectronic with CO.^42,59^

The relative intensities of [C_5_H_3_O_7_Ru]^+^ and [C_5_H_3_O_8_Ru]^−^, i.e., the ions with the highest *m*/*z* and with significant intensity, were higher using the confined setup of the DART (see Tables S7 and S8), wherein [C_5_H_3_O_7_Ru]^+^ even became the most abundant in the spectrum. Much more convincingly than for PVG of W, this indicates that reduced diffusion of ambient air into the DART stream results in less pronounced decarbonylation.

The above experiments suggest that the volatile species of Ru is mononuclear (i.e., with only one Ru atom) and contains 5 CO groups, so it is likely Ru­(CO)_5_. This carbonyl is not commercially available and quite elusive as it is extremely sensitive to heat and light.^39^ It rapidly converts to crystalline Ru_3_(CO)_12_. This solid compound is actually the best-known carbonyl of Ru, stable under ambient conditions, and easily available because it is used in many applications and as a starting compound to synthesize other Ru complexes. Therefore, for comparison, the analysis of the headspace of solid Ru_3_(CO)_12_ was attempted using the open setup of the DART during PVG from 0.01 M HCOOH. When 150 μL of the headspace gas was injected into the GLS II, absolutely no ions emerged, and the same was true when it was injected directly into the DART stream. When the glass vial containing a solid Ru_3_(CO)_12_ was heated to 125 °C on the hot plate, ions characteristic of Ru_3_(CO)_12_ were recorded, but only when the headspace sample was introduced by a syringe directly to the DART stream. The higher signal intensities were obtained by increasing the temperature of the N_2_ discharge gas from 150 °C (optimal in combination with PVG) to 350 °C. This confirms that the volatility of Ru_3_(CO)_12_ is very low. The DART-HRMS spectrum obtained in the positive ion mode using 350 °C of N_2_ discharge gas is presented in Figure S5A.

There were only two main ions detected in the positive ion mode, [C_12_HO_12_Ru_3_]^+^ (100%) at 642.66 *m*/*z* and [C_11_HO_11_Ru_3_]^+^ (55%) and at 614.67 *m*/*z*, corresponding to [Ru_3_(CO)_12_+H]^+^ and [Ru_3_(CO)_11_+H]^+^. The observed isotopic pattern (see enlargement in Figure S5B) clearly indicates the presence of three Ru atoms and correlates very well with the theoretical one modeled at a resolution of ≈65000, characteristic of HRMS measurements at 640 *m*/*z*. This spectrum is thus completely different from that measured with PVG of Ru from 0.01 M HCOOH (Figure 2A and Table S5). The DART-HRMS spectra of Ru_3_(CO)_12_ obtained in the negative ion mode were not very informative because the dominant ions were [O_3_Ru]^−^ (100%) and [O_4_Ru]^−^ (94%) and many ions with 2–3 Ru atoms emerged in the range 285–665 *m*/*z* but with no distinct maximum.

The very similar ions to those depicted in Figure 2A,B were detected with the open setup and same DART and HRMS conditions when the PVG was conducted with 0.005 M HCOONa without any mediator.^15^ Briefly, [C_3_H_3_O_5_Ru]^+^ amounted to 100%, [C_4_H_3_O_6_Ru]^+^ to 71%, [C_3_H_5_O_6_Ru]^+^ to 56%, [C_4_HO_5_Ru]^+^ to 35%, etc., and [C_5_H_3_O_7_Ru]^+^ to 10%; while [O_3_Ru]^−^ to 100%, [O_4_Ru]^−^ to 41%, [C_3_HO_4_Ru]^−^ to 34%, [HO_3_Ru]^−^ to 31%, [C_2_HO_3_Ru]^−^ to 19%, [C_3_H_3_O_5_Ru]^−^ to 17%, etc., and [C_5_H_3_O_8_Ru]^−^ to 7%.

The interrogation of the gas phase was quite challenging for the “analytical” PVG conditions^9^ using 8 M HCOOH (10Co/25Cd), providing a PVG efficiency of nearly 30%. Unlike Cd^2+^, Co ions are converted to volatile species (Co­(CO)_4_H) during PVG,^28^ which is transported into the DART with the volatile species of Ru. In a first approximation to mimic analytical conditions, an alternative medium was selected: 8 M HCOOH (250Cd), which provided a PVG efficiency of only 0.5%, but was sufficient for this purpose. The ions detected in both positive and negative ion modes are listed in Tables S9 and S10. As in the case of PVG using 0.01 M HCOOH (10Cd) or 0.005 M HCOONa, the same major ions were detected with this photochemical medium. The most abundant ions in the positive ion mode were [C_3_H_3_O_5_Ru]^+^ (100%) and [C_4_H_3_O_6_Ru]^+^ (67%), while [C_5_H_3_O_7_Ru]^+^ had a relative intensity of 22%. The most abundant ions in the negative ion mode were [O_3_Ru]^−^ (100%) and [C_3_HO_4_Ru]^−^ (45%), while the relative intensity of [C_5_H_3_O_8_Ru]^−^ was around 8%. This is also evidence that the concentration of HCOOH in the photochemical medium does not significantly affect the identities of the detected ions, e.g., by formation of ion adducts derived from HCOOH, CO, CO_2_, and H_2_ formed during UV photolysis of HCOOH.

Subsequently, the PVG of Ru was performed using 8 M HCOOH (10Co/25Cd). The main problem was that the full mass spectra obtained were plagued by the presence of a myriad of ions without the characteristic isotopic pattern (monoisotopic ^59^Co) from the cogeneration of volatile Co­(CO)_4_H from the added Co^2+^. Due to the high concentration of Co^2+^ used, polymeric Co ions were observed with high abundances in addition to monomeric ones, in both positive and negative ion modes. For example, the most intense ions in the positive ion mode were [CH_3_O_3_Co]^+^ (141% relative to the most abundant ion containing only Ru as the central atom, i.e., [C_3_H_3_O_5_Ru]^+^) at 121.94 *m*/*z*, [C_2_H_8_O_4_N_2_Co]^+^ (231%) at 182.98 *m*/*z*, [C_2_H_5_O_5_NCo_2_]^+^ (289%) at 240.88 *m*/*z*, [C_2_H_7_O_6_NCo_2_]^+^ (1100%) at 258.89 *m*/*z*, [C_3_H_6_O_6_NCo_2_]^+^ (461%) at 269.89 *m*/*z*, [C_3_H_8_O_7_NCo_2_]^+^ (571%) at 287.90 *m*/*z*, [C_4_H_11_O_9_N_2_Co_2_]^+^ (464%) at 348.91 *m*/*z*, [C_4_H_7_O_9_NCo_3_]^+^ (352%) at 389.81 *m*/*z*, [C_5_H_8_O_11_NCo_3_]^+^ (281%) at 434.81 *m*/*z*, and [C_5_H_11_O_11_N_2_Co_3_]^+^ (244%) at 451.84 *m/z.*

In order to distinguish clearly between the ions originating from the spiked Co^2+^ as the mediator and those from Ru as the analyte, PVG was carried out in such a way that Ru standard prepared in 8 M HCOOH (10Co/25Cd) was injected into the stream of carrier comprising 8 M HCOOH (10Co/25Cd). This ensured that any ions whose intensity increased above the baseline following sample injection could only have come from Ru. The same major ions containing only Ru as the central atom were finally detected in both ion modes as in the case when only Cd^2+^ was used (Table S9 and S10), but using a Ru concentration of 5 mg L^–1^. In addition to these ions, several cluster ions containing both Ru and Co could be identified in the spectra during the same experiment , still respecting the isotopic pattern of Ru, for example, [C_4_H_4_O_8_CoRu]^+^ (155% relative to the most abundant ion containing only Ru as the central atom, i.e., [C_3_H_3_O_5_Ru]^+^) at 340.83 *m*/*z*, [C_4_H_5_O_8_CoRu]^+^ (112%) at 341.84 *m*/*z*, [C_4_H_7_O_8_NCoRu]^+^ (192%) at 357.85 *m*/*z*, [C_4_H_6_O_9_CoRu]^+^ (71%) at 358.84 *m*/*z*, [C_5_H_5_O_8_NCoRu]^+^ (83%) at 367.84 *m*/*z*, [C_5_H_4_O_9_CoRu]^+^ (64%) at 368.82 *m*/*z*, and [C_5_H_6_O_10_CoRu]^+^ (68%) at 386.83 *m*/*z*, and several Ru-Co_2–3_-containing ions at >400 *m*/*z*, but their exact identification became much more difficult. The additional experiments provided a convincing evidence that these ions are produced in the DART and are not derived from mixed metal carbonyl clusters generated during PVG (see the Discussion in the Supporting Information).

### Osmium

With respect to the highest PVG efficiency estimated in our preliminary experiments, a 30% (v/v) HCOOH solution was adopted as the photochemical medium according to the work of Yang et al.^23^ Although the authors found a synergistic effect of added Co^2+^ and Cd^2+^ (50 and 20 mg L^–1^, respectively), only 20 mg L^–1^ Cd^2+^ was chosen as the mediator for the first experiment due to significant cogeneration of Co­(CO)_4_H and potential problems with overloading and aggregation with volatile species of Os, as described above for Ru. Despite the low PVG efficiency under these PVG conditions (≈1%), clear DART-HRMS spectra were obtained in the positive and negative ion modes from 1 mg L^–1^ Os (Figure 3A,B and Tables S11 and S12).

**Figure 3:**
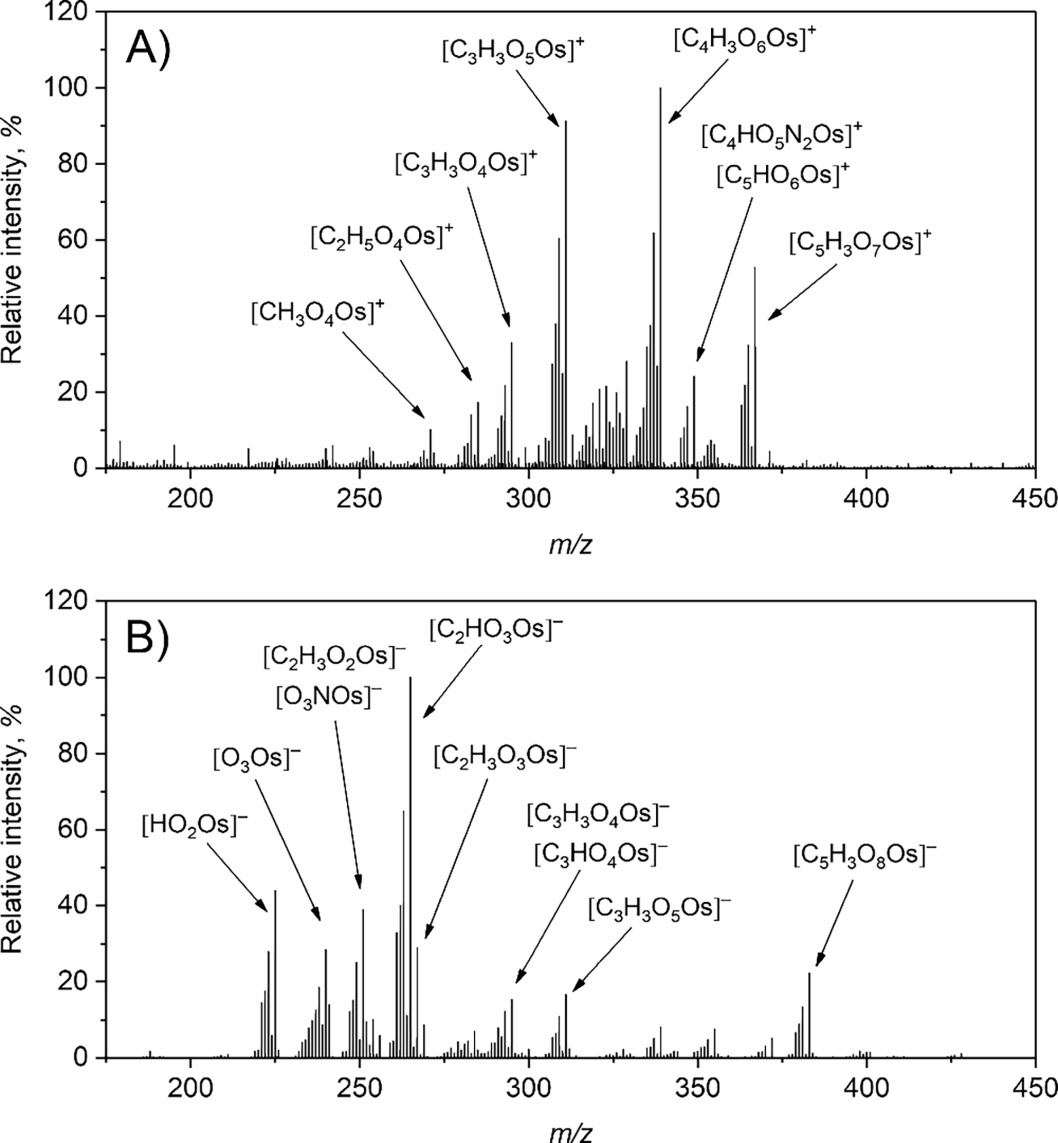
Full scan DART-HRMS spectra obtained in the A) positive and B) negative ion mode during FI-PVG of Os using 30% (v/v) HCOOH (20Cd) and the open setup of the DART. Conditions: 1 mg L^–1^ Os taken for PVG, sample flow rate 2 mL min^–1^; N_2_ discharge gas temperature 150 °C, ion transfer tube temperature 200 °C and 300 °C in the positive and negative ion mode, respectively. Only some ions are labeled, see Tables S11 and S12 for others.

The majority of the ions detected were similar to those detected for Ru, including the N-containing ones. The ions [C_4_H_3_O_6_Os]^+^ (100%) and [C_3_H_3_O_5_Os]^+^ (91%) were the most abundant in the positive ion mode, while [C_5_H_3_O_7_Os]^+^ was the ion with the highest *m*/*z* and significant relative intensity (53%). In the negative ion mode, the most abundant ion was [C_2_HO_3_Os]^−^ (100%), while [C_5_H_3_O_8_Os]^−^, i.e., the last significant ion in the spectrum, amounted to 22% relative intensity. The ions [C_5_H_3_O_7_Os]^+^ and [C_5_H_3_O_8_Os]^−^ were clearly the most abundant when measurements were performed with the confined setup of the DART (Tables S13 and S14). In view of the great similarity of the ions produced from volatile species of Os to those of Ru, it can be assumed that both volatile species have the same structure, with one central atom and five CO groups, meaning that Os­(CO)_5_ is most likely the volatile species generated under these strongly reductive PVG conditions.

When Co^2+^ and Cd^2+^ were used as mediators, the same difficulties with the detection of Os ions as described above for Ru were encountered, with many peaks from Co­(CO)_4_H and aggregated Os-Co ions complicating the evaluation. The same approach of injecting Os standard prepared in 30% (v/v) HCOOH (10Co/20Cd) into the stream of the photochemical medium containing the same concentrations of Co^2+^ and Cd^2+^ had to be used and the major ions (i.e., [C_4_H_3_O_6_Os]^+^ and [C_3_H_3_O_5_Os]^+^) and also [C_5_H_3_O_7_Os]^+^ were finally detected. However, using 50Co/20Cd, no Os-containing ions were detected in both ion modes due to overloading of the DART with Co.

For the photochemical media with no mediator and comprising only 2% (v/v) HCOOH,^4^ the same most abundant ions (listed in Tables S11 and S12) were detected using 5 mg L^–1^ Os in the sample, wherein the results in the negative ion mode were more convincing. The reason for this was the low PVG efficiency (less than 1%) using only HCOOH in the medium and the intensities in the negative ion mode were generally an order of magnitude higher than those in the positive ion mode.

For comparison, analysis of the headspace above solid Os_3_(CO)_12_ was attempted during PVG from 30% (v/v) HCOOH (blank) and very similar behavior was encountered as described for Ru_3_(CO)_12_. Briefly, when 150 μL of the headspace was withdrawn at ambient temperature and injected into the GLS II, absolutely no ions appeared. The same was true when the headspace was injected directly into the stream exiting the DART heated to 350 °C. The ions [C_12_HO_12_Os_3_]^+^ (100%) at 912.83 *m*/*z* and [C_11_HO_11_Os_3_]^+^ (11%) at 884.84 *m*/*z* emerged when the vial with solid Os_3_(CO)_12_ was heated to 125 °C and the headspace was introduced directly to the DART stream (Figure S6A). These ions most probably correspond to [Os_3_(CO)_12_+H]^+^ and [Os_3_(CO)_11_+H]^+^. As in the case of Ru_3_(CO)_12_, the observed isotopic pattern (Figure S6B) clearly indicates the presence of three Os atoms and correlates very well with the theoretical one modeled at a resolution of ≈62000, characteristic of HRMS measurements at 910 *m*/*z*. In contrast to Ru_3_(CO)_12_, the DART-HRMS measurements in the negative ion mode were more useful for the identification of Os_3_(CO)_12_ due to the appearance of [Os_3_(CO)_12_+OH]^−^ (27% relative to the most abundant ion, i.e., [O_3_NOs]^−^) at 928.83 *m*/*z* and [Os_3_(CO)_10_+OH]^−^ (26%) at 872.84 *m*/*z* (not shown). In addition to them, a large number of other Os_1–3_-containing ions in the ranges 220–383 *m*/*z*, 480–665 *m*/*z*, and 785–985 *m*/*z* appeared due to strong fragmentation and oxidation.

Oxidative PVG conditions (5% (v/v) HNO_3_, DIW, and 1% (v/v) H_2_O_2_)^4–6^ were also interrogated by DART-HRMS. No ions were detected in the positive ion mode, while mainly three major ions were detected in the negative ion mode for all three photochemical media, namely [O_3_Os]^−^, [O_3_NOs]^−^, and [O_4_Os]^−^, with some differences in their relative abundances (details given in the Supporting Information, Figure S7A,B,C and Tables S15, S16, and S17). All of these media thus clearly result in OsO_4_ as the volatile species, in agreement with the previous expectations^4–6^ and recent GC-MS evidence.^33^ The fact that ions resulting from OsO_4_ are detected only in the negative ion mode may be due to the proton affinity of OsO_4_ (676.9 kJ mol^–1^), which is significantly lower than that of water (691 kJ mol^–1^) and thus unsuitable for proton transfer reaction, and the high ionization energy (12.35 eV), which excludes Penning ionization by N_2_* and charge transfer by NO^+^ (9.26 eV) and O_2_^+•^ (12.06 eV) produced in the N_2_ discharge gas.

The last PVG conditions that remained to be investigated were 1% (v/v) CH_3_COOH (50Fe), with which de Oliveira et al.^4^ reported the highest PVG efficiency. Although their optimum irradiation time (≈22 s), resulting from the sample flow rate of 2 mL min^–1^, was used in this work, low PVG efficiency of around 2.6% was achieved under these PVG conditions. Surprisingly, absolutely no ions appeared in the positive ion mode and the ions detected in the negative ion mode (Figure S8A and Table S18) were very similar to those obtained during PVG from DIW (Figure S7B and Table S16), during the first few injections of the sample and when the PVG apparatus was clean. This result suggests OsO_4_ as the volatile species again. After 30 min of the PVG with 1% (v/v) CH_3_COOH, several C-containing ions appeared with low abundances, still only in the negative ion mode: [C_2_H_3_O_4_Os]^−^ at 282.97 *m*/*z*, [C_2_H_2_O_5_Os]^−^ at 297.95 *m*/*z*, [C_2_H_2_O_6_Os]^−^ at 313.95 *m*/*z*, [C_4_H_5_O_7_Os]^−^ at 356.97 *m*/*z*, [C_7_H_9_O_6_Os]^−^ at 381.00 *m*/*z*, and [C_6_H_7_O_7_Os]^−^ at 382.98 *m*/*z*. Their intensities started to gradually increase, but especially of [C_4_H_5_O_7_Os]^−^, [C_7_H_9_O_6_Os]^−^, and [C_6_H_7_O_7_Os]^−^, which reached up to around 50% intensity relative to [O_3_NOs]^−^ after 60 min (Figure S8B and Table S19). Interestingly, none of these major ions was detected during PVG from 30% (v/v) HCOOH using both DART setups (c.f. Tables S12 and S14 to S19). The additional experiments, detailed in the Discussion in the Supporting Information, showed that only [C_7_H_9_O_6_Os]^−^ and [C_6_H_7_O_7_Os]^−^ can be attributed to a distinct volatile species. The other C-containing ions, including [C_4_H_5_O_7_Os]^−^, are associated with generated OsO_4_ and apparently originate in the DART or during transport in the gas phase by reactions of OsO_4_ with reducing components of the gas phase derived from the UV photolysis of CH_3_COOH (CO, CO_2_, CH_4_, and C_2_H_6_).^3^ MS^2^ experiments using [C_7_H_9_O_6_Os]^−^ and [C_6_H_7_O_7_Os]^−^ as the precursor ions indicate that the volatile species contains CO group(s), possibly H_2_, and also acetate or more likely methyl group(s) (see the Discussion in the Supporting Information). The full structure of this compound is not completely clear and identification could not be further advanced due to insufficient PVG efficiency and resulting low ion intensities.

## Conclusion

It has been demonstrated that DART ionization induces significant structural changes in volatile metal carbonyls, which can be characterized by severe fragmentation, oxidation, hydration, and formation of various adducts derived from N_2_ used as the discharge gas. Such changes may, to some extent, be related to a lower stability of volatile metal carbonyls and, in particular, their ions under ambient conditions, making the direct identification of unknown volatile metal carbonyls generated by PVG not entirely straightforward. Nevertheless, considering the use of HCOOH in the photochemical medium, which can only give rise to certain volatile metal carbonyl/hydride species, and respecting the 18-valence electron rule required for thermodynamically stable transition metal compounds, the identification of volatile carbonyls of Ru and Os generated by PVG can be advanced. The number of central metal atoms as well as CO groups bound to the central atom is easily deduced from the detected ions in both positive and negative ion modes and even more convincingly using the confined setup of the DART wherein penetration of O_2_ from the ambient atmosphere is limited. The DART-HRMS spectra obtained with HCOOH-based media and in the presence of metal mediators suggest that the volatile species of Ru and Os are mononuclear, contain five CO groups, and are therefore assigned as Ru­(CO)_5_ and Os­(CO)_5_. Obviously, their spectra are significantly different from those obtained from headspace sampling of volatile species above solid Ru_3_(CO)_12_ or Os_3_(CO)_12_, which were taken as examples of the most common and stable Ru and Os carbonyls. Moreover, it was demonstrated that these trinuclear carbonyls lack sufficient volatility required for their efficient transport from the generator to the detector in the gas phase. A more detailed discussion can be found in the Supporting Information.

The same volatile product, Ru­(CO)_5_, was identified regardless of whether dilute HCOOH or concentrated HCOOH was present in the photochemical medium, demonstrating truly reductive PVG conditions even for dilute HCOOH media that were previously reported to provide high PVG efficiency for some analytes.^15^ DART-HRMS was also able to detect OsO_4_ as the volatile species generated not only under strictly oxidative conditions, but surprisingly also using dilute CH_3_COOH. This medium appears to be oxidative, but also reductive to some extent, as some distinct volatile carbonyl/hydrido/methyl (or acetato) species with different physical and chemical properties were tentatively identified.

DART-HRMS identification of volatile 3d-transition metal carbonyls, such as Fe­(CO)_5_, may encounter some difficulties. These issues are partially related to the more pronounced clustering of generated ions and the formation of dimeric and trimeric ions, which occurs at relatively low concentrations of metal carbonyls in the gas phase (see the Supporting Information). However, based on the data for W, Ru, and Os, it appears that DART-HRMS has great potential for identifying other hitherto unknown volatile metal carbonyls generated by PVG, mainly of heavier elements (e.g., Ir, Nb, Re, Rh, and Ta),^11,15,17,60–62^ whose low stability probably precludes the successful use of GC-MS. For example, ongoing experiments devoted to identification of volatile carbonyls of Ir and Rh, both of which have an odd number of valence electrons, seem promising.

Knowing the identities of the compounds and their corresponding physical and chemical properties, is critical to the further development of the PVG technique. Some of these properties may be found in older literature for newly identified compounds.^38,40,41,43^ However, others, such as thermal lability and decomposition rate under the gas-phase conditions resulting from PVG, will need to be determined. This can be, for example, crucial when considering the implementation of additional online preconcentration procedures, such as cryotrapping, to improve limits of detection down to ppq levels. Lastly, the successful identification of mononuclear Ru and Os carbonyls concludes the whole process of developing new synthesis routes. This increases the likelihood that their PVG will be used in other scientific fields in the future, e.g., in situ preparation of precursors or catalysts in organic and organometallic synthesis or chemical vapor deposition.

## Supplementary Material



## Notes

Chemicals and analytical standards used; details of PVG, DART-HRMS, and ICPMS instrumentation, and operating parameters; optimization of DART and HRMS parameters; preliminary experiments using PVG of W and Fe; tables summarizing the Ru and Os ions detected in positive and negative ion modes during FI-PVG using different photochemical media and DART configurations; DART-HRMS spectra obtained from the headspace sampling of solid Ru_3_(CO)_12_ and Os_3_(CO)_12_; details of the experiments focused on PVG of Os using oxidative conditions and dilute CH_3_COOH; discussion of the results; additional experiments devoted to the formation of mixed metal carbonyl ions and to PVG using dilute CH_3_COOH medium (PDF)
