# Supplementary material for: Identification of Photochemically Generated Volatile Species of Ruthenium and Osmium Using Direct Analysis in Real Time Mass Spectrometry
Source: Anal Chem. 2025 Jul 28;97(30):16593–601. doi: 10.1021/acs.analchem.5c02929 (PMC12332834; doi:10.1021/acs.analchem.5c02929)
Supplement: Supplementary file 1 [file ac5c02929_si_001.pdf]

Supporting Information

Identification of Photochemically Generated Volatile Species of Ruthenium and Osmium Using Direct Analysis in Real Time Mass Spectrometry

Ignacio Machado,[a,b] Beatrice Campanella,[a,c] Zhendong Lyu,[a,d] and Stanislav Musil[a,]*

[a] Institute of Analytical Chemistry of the Czech Academy of Sciences, Veveří 97, 602 00 Brno, Czech Republic

[b] Universidad de la República, Faculty of Chemistry, Gral. Flores 2124, Montevideo, Uruguay

[c] National Research Council, Institute of Chemistry of Organometallic Compounds, via Giuseppe Moruzzi 13, 56124 Pisa, Italy

[d] Charles University, Faculty of Science, Department of Analytical Chemistry, Hlavova 8, 128 43 Prague, Czech Republic

* Corresponding author; E-mail: stanomusil@biomed.cas.cz (S. Musil)

TABLE OF CONTENTS

Chemicals and analytical standards used S-4

Details of photochemical vapor generator S-4

Figure S1. FI-PVG setup coupled to DART-HRMS S-5

Details of DART-HRMS and operating parameters S-5

Figure S2. Confined setup of DART for coupling to FI-PVG S-6

Details of ICPMS S-6

Table S1. ICPMS parameters for coupling with PVG S-7

Experiments dealing with optimization of DART and HRMS parameters S-8

Preliminary DART-HRMS experiments using PVG of W and Fe S-8

Figure S3A,B. DART-HRMS spectra obtained in the positive and negative ion mode during PVG of W using 40% (v/v) HCOOH (500Cd) and the open setup of the DART S-10

Table S2. Ions detected in the positive ion mode during PVG of W using 40% (v/v) HCOOH (500Cd) and the open setup of the DART S-11

Table S3. Ions detected in the negative ion mode during PVG of W using 40% (v/v) HCOOH (500Cd) and the open setup of the DART S-12

Figure S4. DART-HRMS spectrum obtained in the negative ion mode during PVG of Fe using 30% (m/v) HCOOH and the open setup of the DART S-14

Table S4. Ions detected in the negative ion mode during PVG of Fe using 30% (m/v) HCOOH and the open setup of the DART S-15

Supporting data for ruthenium S-16

Table S5. Ions detected in the positive ion mode during PVG of Ru using 0.01 M HCOOH (10Cd) and the open setup of the DART S-16

Table S6. Ions detected in the negative ion mode during PVG of Ru using 0.01 M HCOOH (10Cd) and the open setup of the DART S-17

Table S7. Ions detected in the positive ion mode during PVG of Ru using 0.01 M HCOOH (10Cd) and the confined setup of the DART S-18

Table S8. Ions detected in the negative ion mode during PVG of Ru using 0.01 M HCOOH (10Cd) and the confined setup of the DART S-19

Figure S5A,B. DART-HRMS spectrum obtained in the positive ion mode from the headspace sampling of preheated solid $Ru_3(CO)_{12}$ introduced directly to the DART and a close-up of the isotopic patterns of two major ions S-20

Table S9. Ions detected in the positive ion mode during PVG of Ru using 8 M HCOOH (250Cd) and the open setup of the DART S-21

Table S10. Ions detected in the negative ion mode during PVG of Ru using 8 M HCOOH (250Cd) and the open setup of the DART S-22
Supporting data for osmium S-23
Table S11. Ions detected in the positive ion mode during PVG of Os using 30% (v/v) HCOOH (20Cd) and the open setup of the DART S-23
Table S12. Ions detected in the negative ion mode during PVG of Os using 30% (v/v) HCOOH (20Cd) and the open setup of the DART S-24
Table S13. Ions detected in the positive ion mode during PVG of Os using 30% (v/v) HCOOH (20Cd) and the confined setup of the DART S-25
Table S14. Ions detected in the negative ion mode during PVG of Os using 30% (v/v) HCOOH (20Cd) and the confined setup of the DART S-25
Figure S6A,B. DART-HRMS spectrum obtained in the positive ion mode from the headspace sampling of preheated solid $Os_3(CO)_{12}$ introduced directly to the DART and a close-up of the isotopic patterns of two major ions S-27
Experiments focused on PVG of Os using oxidative conditions S-28
Figure S7A,B,C. DART-HRMS spectra obtained in the negative ion mode during PVG of Os using 5% (v/v) HNO_3, DIW, and 1% (v/v) H_2O_2 and the open setup of the DART S-29
Table S15. Ions detected in the negative ion mode during PVG of Os using 5% (v/v) HNO_3 and the open setup of the DART S-30
Table S16. Ions detected in the negative ion mode during PVG of Os using DIW and the open setup of the DART S-30
Table S17. Ions detected in the negative ion mode during PVG of Os using 1% (v/v) H_2O_2 and the open setup of the DART S-31
Figure S8A,B. DART-HRMS spectra obtained in the negative ion mode at the beginning and after 60 min of PVG of Os using 1% (v/v) CH_3COOH (50Fe) S-32
Table S18. Ions detected in the negative ion mode at the beginning of PVG of Os using 1% (v/v) CH_3COOH (50Fe) S-33
Table S19. Ions detected in the negative ion mode after 60 min of PVG of Os using 1% (v/v) CH_3COOH (50Fe) S-34
Discussion of the results and additional experiments devoted to the formation of mixed metal carbonyl ions and to PVG using CH_3COOH medium S-35
Figure S9. Comparison of normalized ICPMS signals obtained during PVG of Os using 5% (v/v) HNO_3, 1% (v/v) CH_3COOH (50Fe), and 30% (v/v) HCOOH (10Co/20Cd) S-37
References S-39

EXPERIMENTAL

Chemicals. Deionized water (DIW, <0.2 μS cm^{-1}, Ultrapur, Watrex), formic acid (98%, p.a., Lach-Ner, Czech Republic), acetic acid (99.8%, p.a., Lach-Ner), sodium formate (Sigma-Aldrich), nitric acid (≥65%, semiconductor grade, Sigma-Aldrich), ammonium hydroxide (25%, p.a., Supelco), and hydrogen peroxide (30%, p.a.+, Analytika, Czech Republic) were used for the formulation of various photochemical media. Commercial stock analytical standard solutions of 1000 mg L^{-1} of individual elements were sourced as follows: Ru^{3+} (as $RuCl_3$) in 1 M HCl and W^{6+} in 2% (v/v) KOH from Sigma-Aldrich; Os^{4+} (as $(NH_4)_2OsCl_6$) in 7% (m/m) HCl from Merck; and Fe^{3+} in 1 M HCl from BDH (UK). The following compounds were used as metal ion mediators for PVG: cadmium(II) acetate dihydrate (p.a., Lach-Ner), cobalt(II) acetate tetrahydrate (99.999%, Alfa Aesar), and iron(II) acetate (≥99.99%, Sigma-Aldrich). Stock solutions of metal mediators were prepared by dissolution of these metal acetates in 0.2% (v/v) CH_3COOH and contained 5–10 g L^{-1} of individual metals. The solid metal carbonyls of $W(CO)_6$, $Ru_3(CO)_{12}$, and $Os_3(CO)_{12}$, as well as liquid $Fe(CO)_5$, were sourced from Sigma-Aldrich.

Photochemical Vapor Generator. The photochemical vapor generator (Figure S1) coupled to DART-HRMS was based on the system used in our previous studies,[1–4] with several modifications. All connecting tubing was made of PTFE (i.d. 1 mm) with the exception of the Tygon pump tubing. The core of the system is a low pressure 19 W thin-film flow-through Hg discharge lamp as a photoreactor (Jitian Instruments Co., China). Delivery of a photochemical medium to the photoreactor at 2 mL min^{-1} (corresponding to irradiation time of about 22 s) was undertaken using a peristaltic pump (Reglo Digital, Ismatec). A flow injection (FI) mode was used when the standards and blanks, prepared in the photochemical medium and possibly spiked with selected transition metal mediators, were introduced into a stream of the photochemical medium with the aid of an injection valve (0.5 mL sample volume). The effluent stream from the photoreactor was mixed with a 100 mL min^{-1} flow of Ar carrier prior to gas-liquid separation. Two gas-liquid separators in series (GLS I and II, made from PP centrifuge vials of 15 mL volume) were used in an effort to eliminate/minimize a carryover of droplets of photochemical medium to the DART. The GLS I was described earlier[4] and was cooled in an ice-water bath throughout the experiment. The GLS II was fitted with a customized PTFE cap containing 3 ports to conveniently introduce the gas phase from the GLS I via PTFE tubing (i.d. 2 mm × 40 cm long), to remove the gas phase for on-line DART-HRMS identification, but also allow for simultaneous introduction of the headspace samples of solid or liquid metal carbonyl standards by a Hamilton syringe (up to 500 μL) through a GC septum.

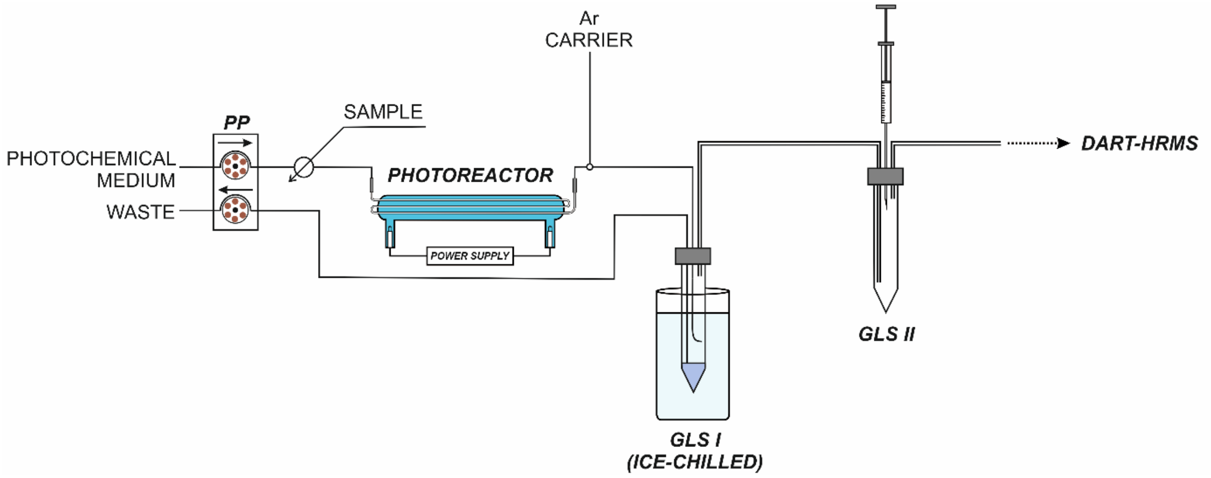

Figure S1. FI-PVG setup coupled to DART-HRMS used for identification of volatile species. GLS – gas-liquid separator, PP – peristaltic pump.

For some experiments, the FI-PVG system was coupled to an inductively coupled plasma mass spectrometer (Agilent 8900 triple quadrupole ICPMS/MS) to estimate the efficiencies of PVG of Os and Fe conducted from various photochemical media. In this case, the GLS II was not used and the gas phase exiting the GLS I was directly introduced into ICPMS. Details of the coupling of PVG to ICPMS and the measurement parameters are described in the "Inductively Coupled Plasma Mass Spectrometry" section of the Supporting Information and in Table S1.

Direct Analysis in Real Time Mass Spectrometry. A DART ion source (model SVPS-300, Ionsense, USA) is equipped with a Vapur Interface for connection to an Orbitrap Exploris 120 high-resolution mass spectrometer (HRMS, Thermo Fisher Scientific, Inc.). The Vapur Interface consists of a 60 mm long ceramic tube (i.d. 4.6 mm) that guides the sampled gas to the orifice of an ion transfer tube of an atmospheric pressure ionization (API) interface. The interior of the Vapur Interface is typically held at reduced pressure by means of a small membrane pump (Vacuubrand). The measurements were conducted using two configurations of the DART when coupled to PVG, i.e., either a standard "open" environment for ionization (Figure 1) or a "confined" setup (Figure S2). In the former configuration, the distance between the tip of the angled ceramic cap of the DART source and the edge of the ceramic capillary of the Vapur Interface was 12 mm. The outlet of the PVG generator was admixed with the stream of N_2 as the discharge gas via a PTFE tube (i.d. 1 mm), wherein the position of the PTFE tube tip was aligned with the horizontal axis of the flow of discharge gas, being approximately 3 mm from the tip of the ceramic cap. In the latter configuration, the ceramic capillary of the Vapur Interface was replaced by the quartz T-tube (i.d. 5 mm, 85 mm long, 35 mm long inlet tube), having the function to protect the ionization

environment from the ambient air as well as guide the ions to the API interface. The end of the shorter (left) arm of the T-tube was pushed tightly onto the tip head of the DART source while the longer (right) arm was attached to the API interface while still keeping a sufficient gap to leak any excessive gas flow. The hose of the rough membrane pump to the Vapur Interface was disconnected in this case.

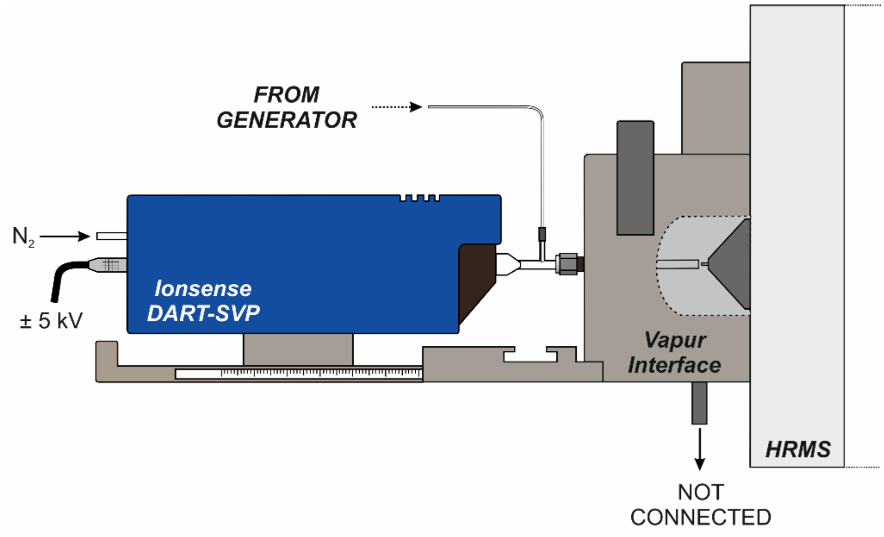

Figure S2. Confined setup of DART for coupling to FI-PVG. The layout of the interior of the Vapur Interface is displayed for clarity in the window defined by the dashed line.

The Orbitrap Exploris 120 mass spectrometer was tuned either in the positive or negative ion mode using a Thermo Scientific OptaMax NG ion source with a low-flow electrospray and the Pierce™ FlexMix™ solution for accurate mass calibration. Alternatively, a one-point mass calibration/check was performed using an internal calibrant delivery (EASY-IC™). Full scan mass spectra were acquired in Chromeleon 7 software at a resolution of 120000 (at 200 *m/z*) in the range 150–2000 *m/z* for PVG of W, 50–750 *m/z* for PVG of Fe, 80–1200 *m/z* for PVG of Ru, and 150–2200 *m/z* for PVG of Os. Unless otherwise stated, the RF lens was set to 70%, the automatic gain control target mode was set to standard, the maximum injection time was set to custom (100 ms), the number of microscans per scan was set to 1, and in-source fragmentation was disabled (cone voltage set to 0 V). The temperature of the ion transfer tube was an investigated parameter. MS^2 measurements with the selected ion precursors were performed with an isolation width of 0.4 *m/z* and normalized collision cell energies of 10–30%.

Inductively Coupled Plasma Mass Spectrometry. In some experiments devoted to PVG of Os or Fe, the FI-PVG system was coupled to an inductively coupled plasma mass spectrometer (Agilent 8900 triple quadrupole ICPMS/MS). The GLS II was not used in this case and the gas

phase exiting the GLS I was introduced into the ultra-high matrix introduction port (UHMI) available with Agilent 8900 ICPMS/MS and located downstream of a Scott double-pass spray chamber. Measurements were performed under “wet” plasma conditions created by simultaneous pneumatic nebulization (PN) of a carrier liquid (2% (m/v) HNO_3) mixed on-line with that of an internal standard (IS, 5 µg L^{-1} Rh and 10 µg L^{-1} Re in 2% (m/v) HNO_3). The liquid carrier channel was equipped with a manual injection valve (0.5 mL sample loop volume) and it was exclusively employed to inject standards of Os or Fe to estimate/determine overall PVG efficiency. The details of this procedure can be found in our previous papers.[2–4] Optimal ICPMS/MS parameters used for parallel coupling of FI-PN and FI-PVG, monitored ions, and their dwell times are summarized in Table S1.

Table S1. Agilent 8900 ICPMS/MS parameters for coupling with FI-PVG

RF power (W)	1550
RF matching (V)	1.3
Sampling depth (mm)	8.0
Nebulizer Ar (mL min^{-1})	1100
Makeup Ar (mL min^{-1})	0
Ar carrier for PVG (mL min^{-1})	100
Carrier liquid/IS flows (mL min^{-1})	0.33/0.08
Spray chamber temperature (°C)	2
He collision gas flow (mL min^{-1})	4.1
Acquisition mode	Time-resolved analysis
Scan type	Single quad
Measured ions (dwell time, s)	$^{103}Rh^+$ (IS, 0.05 s), $^{185}Re^+$ (IS, 0.05 s), $^{56}Fe^+$ (0.1 s), $^{57}Fe^+$ (0.1 s), $^{189}Os^+$ (0.1 s), $^{192}Os^+$ (0.1 s)

RESULTS AND DISCUSSION

Optimization of DART and HRMS Parameters. The effects of some parameters affecting the DART discharge and ion transfer for the HRMS detection were quite similar for the analyte carbonyl species and PVG conditions tested. Some general observations about these parameters are briefly summarized here.

The potential applied to the grid electrode of the DART source should act to remove oppositely charged species (electrons/anions or protons/cations) from the discharge gas. However, no significant changes in the detected ions, their relative abundances, and intensities were observed when the potential was varied from 0 to –350 V and from 0 to +350 V for negative and positive ion modes, respectively. The default values of –350 V and +350 V were thus used throughout.

Conversely, the cone voltage (declustering potential) had a very strong effect, as any applied voltage other than 0 V (5, 10, 35, and 80 V tested) resulted in additional fragmentation (loss of CO groups) and a shift in ion distributions in favor of metal oxide ions, with lower *m/z*. These changes were more pronounced at higher voltages. This may be characteristic of mononuclear metal carbonyls and is consistent with their behavior reported with energy-dependent electrospray ionization mass spectrometry.[5] Therefore, a cone voltage of 0 V (in-source fragmentation switched off) was used during the entire study.

Regarding the RF lens voltage (given in % in the range 0–150%), which is applied to the S-lens to efficiently capture and focus the ions into a tight beam at the exit of the transfer tube, it was found that the ion intensities decreased significantly at values lower than 70%, while severe fragmentation (loss of CO groups and formation of metal oxide ions) emerged at values 100–150%. This suggests a rather low stability of the analyte ions generated in the DART source. An RF value of 70% was chosen as a compromise between the sensitivity and severity of fragmentation. Some degree of these structural changes during ion transport for HRMS detection must still be considered, in addition to more critical changes observed during ionization processes in the DART.

Preliminary DART-HRMS experiments using PVG of W and Fe. Volatile $W(CO)_6$ and $Fe(CO)_5$, either photochemically generated or introduced as standards from a headspace, were employed in order to gain first insights into the ionization of volatile metal carbonyl species in the DART. Volatile $W(CO)_6$ was generated by PVG using 40% (v/v) HCOOH with the addition of 500 mg L^{-1} Cd^{2+} as a mediator,[4] hereafter expressed in the text as 40% (v/v) HCOOH (500Cd). The PVG efficiency is expected to be around 40%, despite the much higher analyte concentrations used in this study. It is important to note that the PVG conditions used for W ensured that the gas

phase was free of volatile species generated from Cd^{2+} as a mediator, because this process is not efficient.[4] PVG of Fe was conducted using either 30% (m/v) HCOOH or 30% (m/v) HCOOH with a pH adjusted to 3.2 using liquid ammonia ($NH_3 \cdot H_2O$). The latter conditions were similar to those that were previously found to be optimal for coupling with atmospheric pressure glow discharge (APGD) and atomic emission spectrometry (AES).[6] The PVG efficiencies for both photochemical media were estimated at a sample flow rate of 2 mL min^{-1} (see "Inductively Coupled Plasma Mass Spectrometry" section) and they were found to be around 1% and 25%, respectively.

A variety of ions was detected and reliably identified in both the positive (Figure S3A and Table S2) and negative ion (Figure S3B and Table S3) modes originating from PVG of 0.2 mg L^{-1} W using the open setup of the DART. The most intense ions in the positive ion mode were $[H_3O_4W]^+$ (100%), $[C_5H_3O_7W]^+$ (53%), and $[C_5HO_6W]^+$ (45%). No $[W(CO)_6]^+$ or $[W(CO)_6+H]^+$ ions formed by simple charge or proton transfer were identified in the spectra, which would provide direct evidence for volatile $W(CO)_6$, although the ionization energy of $W(CO)_6$ (8.0–8.6 eV, NIST database) is favorable and its proton affinity (758 kJ mol^{-1}) is greater than that of water (691 kJ mol^{-1}). Apparently, the DART ionization process at ambient pressure induces significant structural changes characterized by loss of CO groups and their substitution by H_2O and OH^+ (i.e., $[W(CO)_6–xCO+yH_2O+zO+H]^+$). Higher energy collision-induced dissociation (HCD) and measurement in MS^2 mode confirmed the sequential loss of CO (and H_2O) groups from the selected precursor ions $[C_5H_3O_7W]^+$ and $[C_5HO_6W]^+$.

The oxidation and loss of the CO groups was even more pronounced in the negative ion mode (Figure S3B and Table S3) where the most abundant ion was $[HO_4W]^-$ (100%), followed by $[C_3H_3O_8W]^-$ (94%), $[CHO_5W]^-$ (61%), and $[HO_3W]^-$ (33%). In any case, several ions that clearly maintain 6 CO groups are detectable, for example $[C_6H_3O_8W]^+$ (7.4%), which could be $[W(CO)_6+H_2O+OH]^+$. In addition, the relatively highly abundant $[C_5HO_6W]^+$ (45%) indicates the loss of only one CO group and its substitution by OH^+, i.e., $[W(CO)_6–CO+OH]^+$). The ion $[C_6H_3O_9]^-$ at 402.93 *m/z* in the negative ion mode was detected but with very low intensity (1%).

Several N-containing ions appeared at high abundances in the spectra, such as $[H_4O_3NW]^+$ (15%), $[C_2H_4O_4NW]^+$ (12%), $[C_4H_4O_5NW]^+$ (18%), and $[C_5H_4O_6NW]^+$ (17%). These ions are likely NH_4^+ adducts formed by diffusion of ammonia from the ambient air into the DART or by reaction of reactive species of N_2 with H_2 formed during UV photolysis of HCOOH.[7] These NH_4^+ adducts were confirmed by the experiment in which a vial with 10 mL ammonia (25%) was opened at a distance of 1 m from the DART, which was immediately followed by an increase in their intensities up to 100% relative abundance (for $[C_4H_4O_5NW]^+$ and $[C_5H_4O_6NW]^+$). In the negative ion mode, N-containing ions, such as $[O_6NW]^-$ (6.2%) and $[C_2H_2O_9NW]^-$ (8.2%), are likely

formed by additional NO_2^- or NO_3^- attachment, after abstraction of CO groups, as $[NO_3]^-$ is detected at high intensity in the spectrum at 61.99 *m/z*.

Using headspace injection (25 µL) of $W(CO)_6$ as a standard, either into the GLS II or directly into the N_2 stream of the DART while continuously processing a PVG blank (40% (v/v) HCOOH), the same major ions listed in Tables S2 and S3 were detected in both positive and negative ion modes, respectively. Also, when PVG was disconnected and the headspace was injected into a stream of pure Ar carrier, the same ions appeared, but their distribution somehow shifted toward lighter ions (oxides). This may be due to the fact that PVG with 40% (v/v) HCOOH provides highly reductive conditions in the gas phase, which also contains large amounts of CO, CO_2, and H_2 gases.

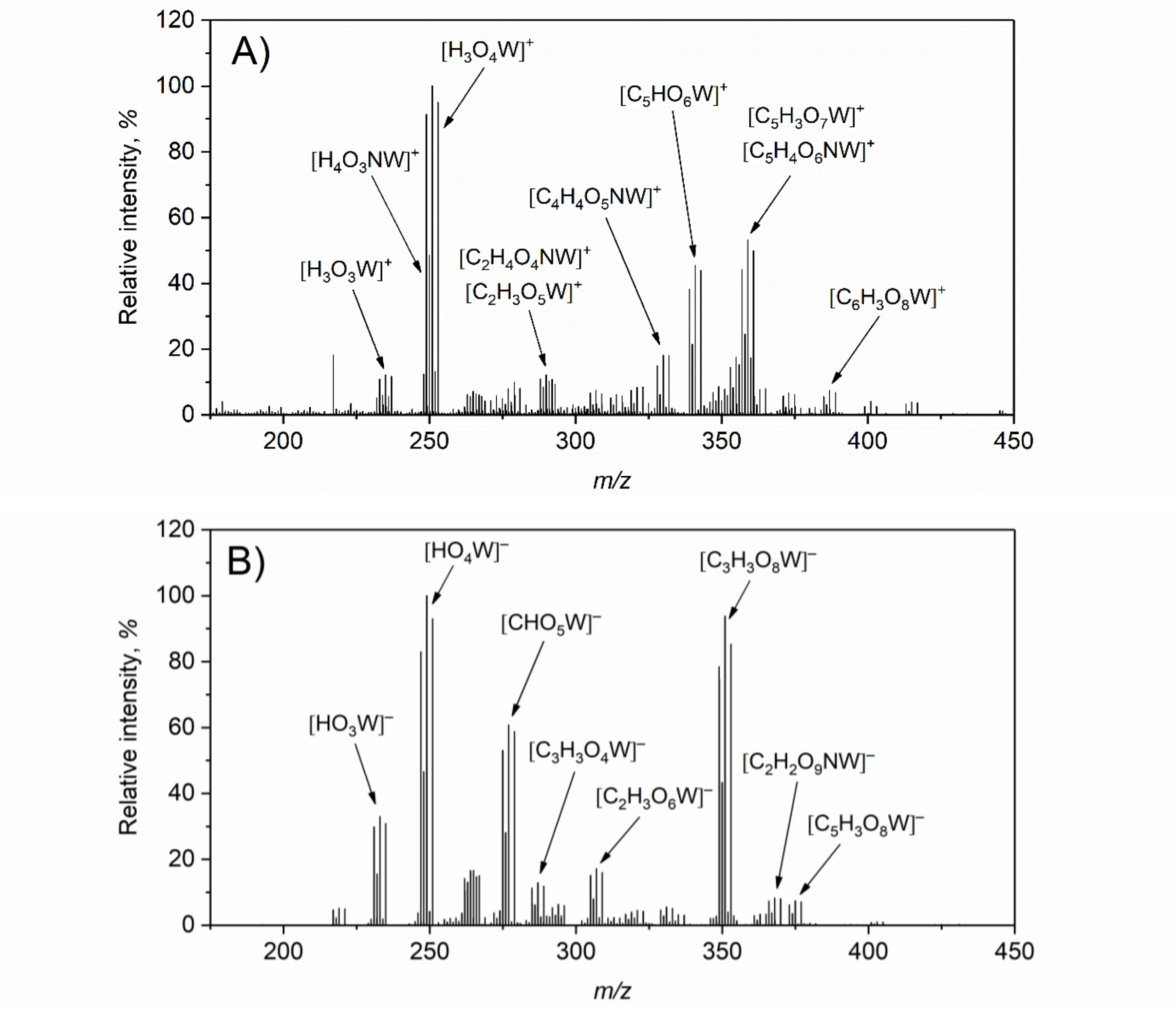

Figure S3. Full scan DART-HRMS spectra obtained in the A) positive and B) negative ion mode during FI-PVG of W using 40% (v/v) HCOOH (500Cd) and the open setup of the DART. Conditions: 0.2 mg L^{-1} W taken for PVG, sample flow rate of 2 mL min^{-1}; N_2 discharge gas temperature 150 °C, ion transfer tube temperature 300 °C and 200 °C in the positive and negative ion mode, respectively. Only some ions are labeled, see Tables S2 and S3 for others.

Table S2. Ions corresponding to ^{184}W with their relative intensities detected in the positive ion mode during FI-PVG of W using 40% (v/v) HCOOH (500Cd) and the open setup of the DART. Conditions: 0.2 mg L^{-1} W taken for PVG, sample flow rate 2 mL min^{-1}; N_2 discharge gas temperature 150 °C, ion transfer tube temperature 300 °C. Ions of relative intensity lower than 5% not shown.

Mass (*m/z*)	Elemental composition	Δ*m/z* (ppm)	Relative intensity[a] (%)
233.9743	H_4O_2NW	–1.19	5.9
**234.9583**	**H_3O_3W**	**–1.33**	**12**
**249.9692**	**H_4O_3NW**	**–1.29**	**15**
**250.9532**	**H_3O_4W**	**–1.43**	**100**
264.9688	CH_5O_4W	–1.35	7.0
265.9403	H_2O_5W	–1.31	6.5
272.9375	C_2HO_4W	–1.46	5.7
278.9719	CH_5O_4NW	–1.30	9.8
**289.9639**	**$C_2H_4O_4NW$**	**–1.68**	**12**
**290.9480**	**$C_2H_3O_5W$**	**–1.66**	**10**
306.9668	$C_2H_5O_5NW$	–1.13	7.4
313.9640	$C_4H_4O_4NW$	–1.30	6.1
320.9949	$C_4H_9O_5W$	–1.50	8.4
**329.9588**	**$C_4H_4O_5NW$**	**–1.49**	**18**
**340.9272**	**C_5HO_6W**	**–1.58**	**45**
349.0262	$C_6H_{13}O_5W$	–1.47	8.6
**354.9429**	**$C_6H_3O_6W$**	**–1.40**	**18**
**357.9538**	**$C_5H_4O_6NW$**	**–1.30**	**17**
**358.9377**	**$C_5H_3O_7W$**	**–1.45**	**53**
363.0418	$C_7H_{15}O_5W$	–1.41	7.7
372.9534	$C_6H_5O_7W$	–1.48	6.6
386.9327	$C_6H_3O_8W$	–1.26	7.4

[a] Signal intensity of the most abundant ion corresponds to 3.5×10^5 counts

Table S3. Ions corresponding to ^{184}W with their relative intensities detected in the negative ion mode during FI-PVG of W using 40% (v/v) HCOOH (500Cd) and the open setup of the DART. Conditions: 0.2 mg L^{-1} W taken for PVG, sample flow rate 2 mL min^{-1}; N_2 discharge gas temperature 150 °C, ion transfer tube temperature 200 °C. Ions of relative intensity lower than 5% not shown.

Mass (*m/z*)	Elemental composition	Δ*m/z* (ppm)	Relative intensity[a] (%)
218.9646	H_3O_2W	–0.99	5.1
**232.9438**	**HO_3W**	**–0.99**	**33**
**248.9388**	**HO_4W**	**–0.50**	**100**
**263.9259**	**O_5W**	**–0.77**	**17**
**264.9337**	**HO_5W**	**–0.83**	**17**
**276.9337**	**CHO_5W**	**–0.58**	**61**
**286.9543**	**$C_3H_3O_4W$**	**–0.99**	**13**
293.9238	O_6NW	–0.73	6.2
**306.9442**	**$C_2H_3O_6W$**	**–0.70**	**17**
330.9443	$C_4H_3O_6W$	–0.56	5.5
**350.9338**	**$C_3H_3O_8W$**	**–1.24**	**94**
367.9240	$C_2H_2O_9NW$	–1.22	8.2
374.9338	$C_5H_3O_8W$	–1.32	7.4

[a] Signal intensity of the most abundant ion corresponds to 9.7×10^6 counts

Using the confined setup of the DART, the ion intensities were generally almost an order of magnitude higher. There was a noticeable increase in the relative abundances of some heavier ions, especially in the positive ion mode, although $[H_3O_4W]^+$ remained the most abundant. For example, the relative abundance of $[C_6H_3O_8W]^+$ increased 2.5-fold to nearly 19%. This indicates that the reduced diffusion of ambient air into the DART stream results in less pronounced decarbonylation of $W(CO)_6$ and its generated ions, which are labile and tend to oxidize rapidly. The N-containing ions, such as $[C_4H_4O_5NW]^+$ and $[C_5H_4O_6NW]^+$, were still highly abundant, with relative abundances of 59% and 72%, respectively, indicating that these NH_4^+ adducts do not originate solely from diffusion of ammonia from the ambient atmosphere.

Due to its structural similarity to the potential mononuclear carbonyls of Ru and Os, i.e., $Ru(CO)_5$ and $Os(CO)_5$, the ionization in the N_2 DART was also briefly examined using $Fe(CO)_5$ and the open setup of the DART. Unlike $W(CO)_6$, the DART-HRMS spectra originating from the

introduction of $Fe(CO)_5$ were unfortunately not very useful in the positive ion mode. Using 30% (m/v) HCOOH as the photochemical medium and 2.5 mg L^{-1} Fe, myriads of ions were detected and it was impossible to reliably identify the majority of them, except for the most abundant: ($[C_2H_4O_5Fe]^+$ (100%) at 163.94 *m/z*, $[C_2H_6O_6Fe]^+$ (39%) at 181.95 *m/z*, $[C_3H_6O_5Fe]^+$ (38%) at 177.96 *m/z*, $[C_2H_5O_4NFe]^+$ (20%) at 162.96 *m/z*, $[C_2H_5O_5Fe]^+$ (15%) at 164.95 *m/z*, $[CH_4O_4Fe]^+$ (14%) at 135.95 *m/z*, $[CH_3O_3Fe]^+$ (13%) at 118.94 *m/z*, and $[C_4H_8O_6Fe]^+$ (10%) at 207.97 *m/z*. The main obstacle to properly identify other, less abundant ions and possibly with 5 carbon atoms, was the "inconvenient" isotopic pattern of Fe (the ^{56}Fe isotope accounts for 91.8%), which made distinguishing the ions´ signatures in the spectra difficult. Additionally, the absolute signal intensities of the Fe-containing ions were substantially lower than those of the background ions. This created the potential for their mutual association after ionization in the DART and the formation of artifact ions. Simply increasing the Fe concentration in the introduced standards for PVG or using the photochemical media characterized by higher PVG efficiency (pH adjusted to 3.2) did not fully resolve this issue because the Fe-containing ions began to aggregate and form dimeric and trimeric ions preferentially, e.g., $[C_3H_5O_8Fe_2]^+$ at 280.87 *m/z*, $[C_4H_5O_9Fe_2]^+$ at 308.86 *m/z*, and $[C_5H_5O_{11}Fe_3]^+$ at 408.79 *m/z*. Similar clustering upon DART ionization was also identified for the volatile species of Co when its PVG was conducted from 30% (m/v) HCOOH (pH = 3.2) at concentrations ≥50 µg L^{-1} (data not shown), and especially when it was used as the mediator at ppm levels during PVG of Ru (see the "Ruthenium" section of the main article for details), while it was not encountered to such an extent for W at concentrations up to 1 mg L^{-1}. This behavior may not be due to higher concentrations of volatile metal carbonyls in the DART, because the PVG efficiencies were not markedly higher than those for W. Hence, it is likely characteristic of the 3d-transition metal carbonyls compared to carbonyls with heavier central atoms, such as W, Ru, and Os.

Conversely, the spectra obtained in the negative ion mode during PVG of Fe using 30% (m/v) HCOOH were much clearer and easier to evaluate, and the ion intensities were sufficiently high (Figure S4 and Table S4). The most abundant ions were $[C_4H_4O_8Fe]^-$ (100%), $[C_2H_2O_4Fe]^-$ (72%), and $[C_2H_3O_4Fe]^-$ (51%). There was one significantly abundant ion in the spectrum that contained 5 carbon atoms, thus likely with 5 CO groups, and it was $[C_5H_6O_7Fe]^-$ (11%). It was detected at 233.95 *m/z* and was fully separated from the adjacent $[C_4H_4O_8{}^{54}Fe]^-$ at 233.93 *m/z*. The relative abundance of other ions with 5 carbon atoms, such as $[C_5H_5O_7Fe]^-$, $[C_5H_6O_8Fe]^-$, and $[C_5H_4O_9Fe]^-$ was typically ≤1%. The DART-HRMS spectrum obtained by the headspace injection of 5 µL of $Fe(CO)_5$ in the negative ion mode was identical to that in Figure S4. The same was true for the spectrum obtained during experiments using 30% (m/v) HCOOH (pH = 3.2) as the photochemical

medium and 0.1 mg L^{-1} Fe. The pronounced formation of dimeric and trimeric ions in the DART, as observed in the positive ion mode, was observed in the negative ion mode at Fe concentrations of ≥0.2 mg L^{-1}, e.g., $[C_4H_4O_9Fe_2]^-$ at 307.86 *m/z* and $[C_5H_5O_{11}Fe_2]^-$ at 352.85 *m/z*.

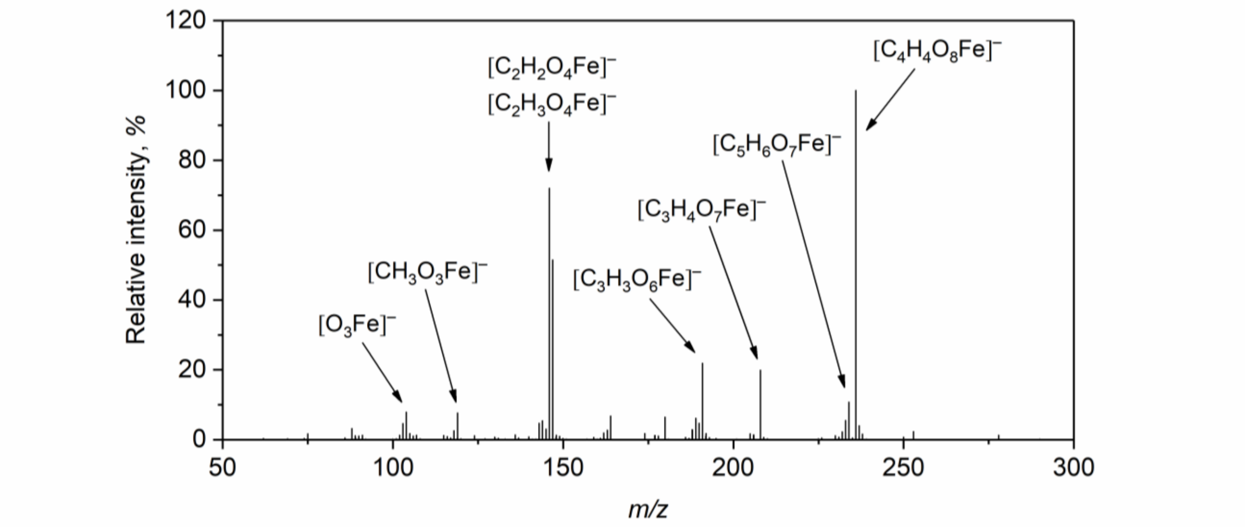

Figure S4. Full scan DART-HRMS spectra obtained in the negative ion mode during FI-PVG of Fe using 30% (m/v) HCOOH and the open setup of the DART. Conditions: 2.5 mg L^{-1} Fe taken for PVG, sample flow rate of 2 mL min^{-1}; N_2 discharge gas temperature 50 °C, ion transfer tube temperature 300 °C.

Table S4. Ions corresponding to ^{56}Fe with their relative intensities detected in the negative ion mode during FI-PVG of Fe using 30% (m/v) HCOOH and the open setup of the DART. Conditions: 2.5 mg L^{-1} Fe taken for PVG sample flow rate 2 mL min^{-1}; N_2 discharge gas temperature 50 °C, ion transfer tube temperature 300 °C. Ions of relative intensity lower than 5% not shown.

Mass (*m/z*)	Elemental composition	Δ*m/z* (ppm)	Relative intensity[a] (%)
103.9201	O_3Fe	–1.68	7.9
118.9435	CH_3O_3Fe	–1.42	7.6
**145.9307**	**$C_2H_2O_4Fe$**	**–0.54**	**72**
**146.9385**	**$C_2H_3O_4Fe$**	**–0.71**	**51**
163.9412	$C_2H_4O_5Fe$	–0.82	6.7
179.9361	$C_2H_4O_6Fe$	–0.99	6.4
188.9490	$C_4H_5O_5Fe$	–0.95	6.1
**190.9283**	**$C_3H_3O_6Fe$**	**–0.96**	**22**
**207.9311**	**$C_3H_4O_7Fe$**	**–0.78**	**20**
232.9263	$C_4H_3O_7NFe$	–0.74	5.5
**233.9467**	**$C_5H_6O_7Fe$**	**–0.82**	**11**
**235.9260**	**$C_4H_4O_8Fe$**	**–0.63**	**100**

[a] Signal intensity of the most abundant ion corresponds to 2.7×10^6 counts

Ruthenium.

Table S5. Ions corresponding to ^{102}Ru with their relative intensities detected in the positive ion mode during FI-PVG of Ru using 0.01 M HCOOH (10Cd) and the open setup of the DART. Conditions: 1 mg L^{-1} Ru taken for PVG, sample flow rate 2 mL min^{-1}; N_2 discharge gas temperature 150 °C, ion transfer tube temperature 200 °C. Ions of relative intensity lower than 5% not shown.

Mass (*m/z*)	Elemental composition	Δ*m/z* (ppm)	Relative intensity[a] (%)
151.9040	H_2O_3Ru	–1.02	8.1
152.9119	H_3O_3Ru	–0.98	5.0
167.9227	H_4O_3NRu	–1.25	5.3
194.9337	$CH_5O_3N_2Ru$	–0.56	9.0
202.8910	C_3HO_4Ru	–1.45	6.7
**205.9020**	**$C_2H_2O_4NRu$**	**–0.75**	**11**
210.9173	$C_2H_5O_5Ru$	–0.95	5.5
**220.9016**	**$C_3H_3O_5Ru$**	**–0.95**	**40**
**230.8860**	**C_4HO_5Ru**	**–0.95**	**51**
**238.9122**	**$C_3H_5O_6Ru$**	**–0.90**	**42**
**248.8965**	**$C_4H_3O_6Ru$**	**–0.94**	**100**
**256.9228**	**$C_3H_7O_7Ru$**	**–0.81**	**11**
**258.8921**	**$C_4HO_5N_2Ru$**	**–1.03**	**17**
266.9071	$C_4H_5O_7Ru$	–0.71	6.1
**276.8914**	**$C_5H_3O_7Ru$**	**–1.04**	**27**

[a] Signal intensity of the most abundant ion corresponds to 1.1×10^6 counts

Table S6. Ions corresponding to ^{102}Ru with their relative intensities detected in the negative ion mode during FI-PVG of Ru using 0.01 M HCOOH (10Cd) and the open setup of the DART. Conditions: 0.2 mg L^{-1} Ru taken for PVG, sample flow rate 2 mL min^{-1}; N_2 discharge gas temperature 150 °C, ion transfer tube temperature 200 °C. Ions of relative intensity lower than 5% not shown.

Mass (*m/z*)	Elemental composition	Δ*m/z* (ppm)	Relative intensity[a] (%)
133.8946	O_2Ru	–1.10	8.0
**134.9024**	**HO_2Ru**	**–0.98**	**27**
135.9102	H_2O_2Ru	–1.16	5.2
**149.8896**	**O_3Ru**	**–0.48**	**100**
**150.8973**	**HO_3Ru**	**–1.04**	**30**
**165.8844**	**O_4Ru**	**–1.01**	**25**
166.8922	HO_4Ru	–1.03	6.0
**174.8973**	**C_2HO_3Ru**	**–0.90**	**23**
175.9051	$C_2H_2O_3Ru$	–0.92	6.0
**176.9129**	**$C_2H_3O_3Ru$**	**–1.00**	**19**
178.8922	CHO_4Ru	–1.02	5.2
**190.8922**	**C_2HO_4Ru**	**–0.80**	**18**
193.9032	CH_2O_4NRu	–0.47	7.2
**202.8922**	**C_3HO_4Ru**	**–0.80**	**46**
**220.9028**	**$C_3H_3O_5Ru$**	**–0.84**	**25**
247.8899	$C_4H_2O_6Ru$	–0.71	7.4
248.8977	$C_4H_3O_6Ru$	–0.77	6.8
**292.8875**	**$C_5H_3O_8Ru$**	**–0.58**	**11**

[a] Signal intensity of the most abundant ion corresponds to 3.8 × 10^6 counts

Table S7. Ions corresponding to ^{102}Ru with their relative intensities detected in the positive ion mode during FI-PVG of Ru using 0.01 M HCOOH (10Cd) and the confined setup of the DART. Conditions: 0.2 mg L^{-1} Ru taken for PVG, sample flow rate 2 mL min^{-1}; N_2 discharge gas temperature 150 °C, ion transfer tube temperature 200 °C. Ions of relative intensity lower than 5% not shown.

Mass (*m/z*)	Elemental composition	Δ*m/z* (ppm)	Relative intensity[a] (%)
115.9066	NRu	–2.46	6.1
118.9301	H_3NRu	–2.53	6.5
135.9566	H_6N_2Ru	–1.98	5.2
167.9225	H_4O_3NRu	–2.38	6.9
**194.9334**	**$CH_5O_3N_2Ru$**	**–1.94**	**11**
**195.9174**	**CH_4O_4NRu**	**–2.06**	**14**
**205.9018**	**$C_2H_2O_4NRu$**	**–1.96**	**58**
**212.9441**	**$CH_7O_4N_2Ru$**	**–1.42**	**10**
**222.9283**	**$C_2H_5O_4N_2Ru$**	**–1.90**	**86**
**230.8857**	**C_4HO_5Ru**	**–2.16**	**57**
**236.9201**	**$C_3H_5O_5NRu$**	**–2.04**	**72**
**239.9549**	**$C_2H_8O_4N_3Ru$**	**–1.51**	**20**
**248.8963**	**$C_4H_3O_6Ru$**	**–1.99**	**49**
**258.8918**	**$C_4HO_5N_2Ru$**	**–1.96**	**19**
**265.8990**	**$C_4H_4O_7Ru$**	**–1.97**	**19**
**276.8911**	**$C_5H_3O_7Ru$**	**–2.09**	**100**
290.9067	$C_6H_5O_7Ru$	–2.06	5.1
**316.9223**	**$C_8H_7O_7Ru$**	**–2.05**	**13**
322.9482	$C_{11}H_9O_5Ru$	–1.95	5.4

[a] Signal intensity of the most abundant ion corresponds to 7.7×10^6 counts

Table S8. Ions corresponding to ^{102}Ru with their relative intensities detected in the negative ion mode during FI-PVG of Ru using 0.01 M HCOOH (10Cd) and the confined setup of the DART. Conditions: 0.2 mg L^{-1} Ru taken for PVG, sample flow rate 2 mL min^{-1}; N_2 discharge gas temperature 50 °C, ion transfer tube temperature 200 °C. Ions of relative intensity lower than 5% not shown.

Mass (*m/z*)	Elemental composition	Δ*m/z* (ppm)	Relative intensity[a] (%)
133.8946	O_2Ru	–0.65	5.7
**134.9025**	**HO_2Ru**	**–0.54**	**13**
135.9103	H_2O_2Ru	–0.72	5.3
**145.8947**	**CO_2Ru**	**–0.53**	**11**
**146.9025**	**CHO_2Ru**	**–0.63**	**17**
**149.8895**	**O_3Ru**	**–0.75**	**19**
**150.8974**	**HO_3Ru**	**–0.64**	**10**
161.9133	CH_2O_2NRu	–0.88	6.4
163.9052	CH_2O_3Ru	–0.44	8.6
**174.8974**	**C_2HO_3Ru**	**–0.33**	**100**
**175.9052**	**$C_2H_2O_3Ru$**	**–0.52**	**20**
**176.9130**	**$C_2H_3O_3Ru$**	**–0.66**	**33**
189.8845	C_2O_4Ru	–0.46	7.2
**191.9001**	**$C_2H_2O_4Ru$**	**–0.51**	**10**
193.9032	CH_2O_4NRu	–0.37	9.0
**202.8923**	**C_3HO_4Ru**	**–0.35**	**69**
**220.9029**	**$C_3H_3O_5Ru$**	**–0.44**	**28**
237.8930	$C_2H_2O_6NRu$	–0.51	8.7
248.8978	$C_4H_3O_6Ru$	–0.37	7.7
281.8954	$C_4H_4O_8Ru$	–0.41	6.1
**292.8876**	**$C_5H_3O_8Ru$**	**–0.41**	**14**
309.8777	$C_4H_2O_9NRu$	–0.37	5.1

[a] Signal intensity of the most abundant ion corresponds to 1.7×10^7 counts

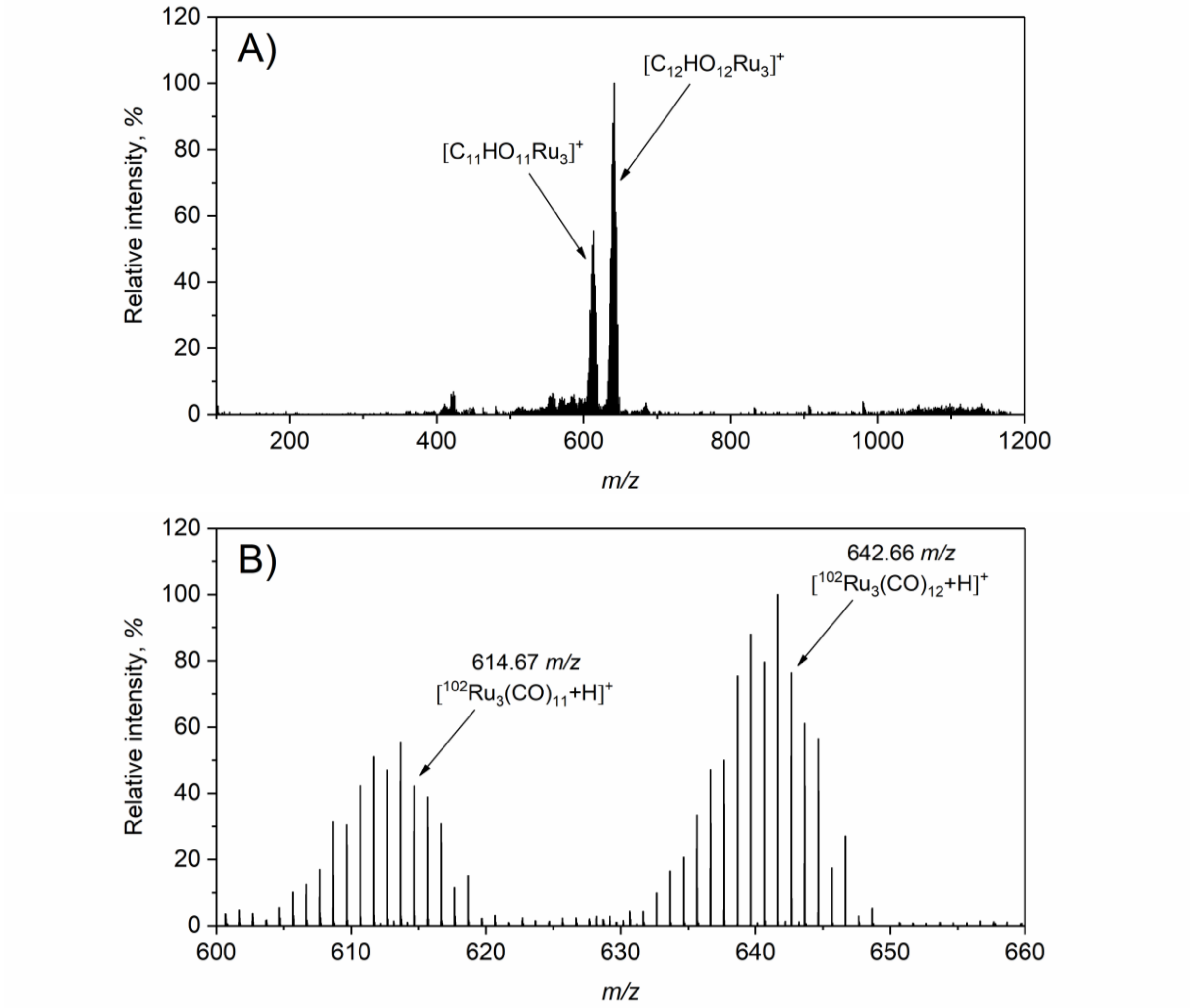

Figure S5. A) Full scan DART-HRMS spectrum obtained in the positive ion mode from the headspace sampling of preheated (125 °C) solid $Ru_3(CO)_{12}$ introduced directly to the DART and B) a close-up of the isotopic patterns of two major ions (600–660 *m/z*) with specified ions containing only ^{102}Ru. Conditions: PVG operated with 0.01 M HCOOH, sample flow rate 2 mL min^{-1}; N_2 discharge gas temperature 350 °C, ion transfer tube temperature 200 °C.

Table S9. Ions corresponding to ^{102}Ru with their relative intensities detected in the positive ion mode during FI-PVG of Ru using 8 M HCOOH (250Cd) and the open setup of the DART. Conditions: 1 mg L^{-1} Ru taken for PVG, sample flow rate 2 mL min^{-1}; N_2 discharge gas temperature 150 °C, ion transfer tube temperature 200 °C. Ions of relative intensity lower than 5% not shown.

Mass (*m/z*)	Elemental composition	Δ*m/z* (ppm)	Relative intensity[a] (%)
151.9041	H_2O_3Ru	–0.89	6.8
152.9119	H_3O_3Ru	–0.85	5.0
198.9173	CH_5O_5Ru	–0.80	5.0
**202.8911**	**C_3HO_4Ru**	**–1.06**	**16**
210.9173	$C_2H_5O_5Ru$	–0.81	6.7
**220.9017**	**$C_3H_3O_5Ru$**	**–0.86**	**100**
**230.8860**	**C_4HO_5Ru**	**–0.95**	**36**
**238.9122**	**$C_3H_5O_6Ru$**	**–0.94**	**45**
**248.8965**	**$C_4H_3O_6Ru$**	**–1.02**	**67**
**258.8921**	**$C_4HO_5N_2Ru$**	**–1.07**	**11**
**276.8914**	**$C_5H_3O_7Ru$**	**–1.08**	**22**
290.9798	$C_8H_{13}O_5Ru$	–1.07	5.6

[a] Signal intensity of the most abundant ion corresponds to 1.2 × 10^6 counts

Table S10. Ions corresponding to ^{102}Ru with their relative intensities detected in the negative ion mode during FI-PVG of Ru using 8 M HCOOH (250Cd) and the open setup of the DART. Conditions: 1 mg L^{-1} Ru taken for PVG, sample flow rate 2 mL min^{-1}; N_2 discharge gas temperature 150 °C, ion transfer tube temperature 200 °C. Ions of relative intensity lower than 5% not shown.

Mass (*m/z*)	Elemental composition	Δ*m/z* (ppm)	Relative intensity[a] (%)
133.8945	O_2Ru	–1.40	7.0
**134.9024**	$\mathbf{HO_2Ru}$	**–1.28**	**23**
**149.8895**	$\mathbf{O_3Ru}$	**–0.88**	**100**
**150.8973**	$\mathbf{HO_3Ru}$	**–1.44**	**28**
**165.8843**	$\mathbf{O_4Ru}$	**–1.37**	**29**
166.8922	HO_4Ru	–1.27	7.1
**174.8973**	$\mathbf{C_2HO_3Ru}$	**–1.19**	**22**
**175.8924**	$\mathbf{CO_3NRu}$	**–1.63**	**11**
**176.9129**	$\mathbf{C_2H_3O_3Ru}$	**–1.29**	**16**
**190.8922**	$\mathbf{C_2HO_4Ru}$	**–1.11**	**18**
193.9031	CH_2O_4NRu	–0.93	7.0
**202.8922**	$\mathbf{C_3HO_4Ru}$	**–1.09**	**45**
203.9000	$C_3H_2O_4Ru$	–1.11	5.2
**220.9027**	$\mathbf{C_3H_3O_5Ru}$	**–1.12**	**20**
237.8930	$C_2H_2O_6NRu$	–0.63	5.3
247.8898	$C_4H_2O_6Ru$	–0.99	7.1
248.8976	$C_4H_3O_6Ru$	–0.93	5.5
292.8875	$C_5H_3O_8Ru$	–0.79	8.0

[a] Signal intensity of the most abundant ion corresponds to 2.0×10^6 counts

Osmium.

Table S11. Ions corresponding to ^{192}Os with their relative intensities detected in the positive ion mode during FI-PVG of Os using 30% (v/v) HCOOH (20Cd) and the open setup of the DART. Conditions: 1 mg L^{-1} Os taken for PVG, sample flow rate 2 mL min^{-1}; N_2 discharge gas temperature 150 °C, ion transfer tube temperature 200 °C. Ions of relative intensity lower than 5% not shown.

Mass (m/z)	Elemental composition	$\Delta m/z$ (ppm)	Relative intensity[a] (%)
241.9850	H_4O_2NOs	–0.60	5.8
252.9534	CHO_3Os	–0.55	5.4
**270.9639**	**CH_3O_4Os**	**–0.57**	**10**
**284.9795**	**$C_2H_5O_4Os$**	**–0.68**	**17**
**294.9639**	**$C_3H_3O_4Os$**	**–0.69**	**33**
298.9951	$C_3H_7O_4Os$	–0.75	5.3
304.9482	C_4HO_4Os	–0.87	7.8
309.9510	$C_3H_2O_5Os$	–0.56	9.1
**309.9750**	**$C_3H_4O_4NOs$**	**–0.01**	**25**
**310.9588**	**$C_3H_3O_5Os$**	**–0.61**	**91**
312.9744	$C_3H_5O_5Os$	–0.76	8.7
**318.9639**	**$C_5H_3O_4Os$**	**–0.51**	**17**
**320.9431**	**C_4HO_5Os**	**–0.81**	**21**
**322.9587**	**$C_4H_3O_5Os$**	**–0.80**	**22**
**325.9459**	**$C_3H_2O_6Os$**	**–0.64**	**20**
327.9615	$C_3H_4O_6Os$	–0.57	7.2
**327.9853**	**$C_3H_6O_5NOs$**	**–0.82**	**10**
**328.9693**	**$C_3H_5O_6Os$**	**–0.71**	**28**
**335.9302**	**C_4O_6Os**	**–0.59**	**25**
**337.9459**	**$C_4H_2O_6Os$**	**–0.59**	**27**
**337.9696**	**$C_4H_4O_5NOs$**	**–0.76**	**22**
**338.9536**	**$C_4H_3O_6Os$**	**–0.75**	**100**
**348.9380**	**C_5HO_6Os**	**–0.61**	**14**
**348.9492**	**$C_4HO_5N_2Os$**	**–0.68**	**24**
353.9408	$C_4H_2O_7Os$	–0.63	7.2
354.9486	$C_4H_3O_7Os$	–0.53	6.1

365.9281	C_4O_7NOs	–0.76	5.5
**366.9485**	**$C_5H_3O_7Os$**	**–0.81**	**53**

[a] Signal intensity of the most abundant ion corresponds to 1.1 × 10^5 counts

Table S12. Ions corresponding to ^{192}Os with their relative intensities detected in the negative ion mode during FI-PVG of Os using 30% (v/v) HCOOH (20Cd) and the open setup of the DART. Conditions: 1 mg L^{-1} Os taken for PVG, sample flow rate 2 mL min^{-1}; N_2 discharge gas temperature 150 °C, ion transfer tube temperature 300 °C. Ions of relative intensity lower than 5% not shown.

Mass (*m/z*)	Elemental composition	Δ*m/z* (ppm)	Relative intensity[a] (%)
223.9516	O_2Os	–1.10	5.8
**224.9595**	**HO_2Os**	**–0.85**	**44**
238.9750	CH_3O_2Os	–1.26	7.3
**239.9465**	**O_3Os**	**–1.01**	**28**
239.9829	CH_4O_2Os	–1.03	6.4
**240.9543**	**HO_3Os**	**–1.06**	**14**
**250.9751**	**$C_2H_3O_2Os$**	**–0.92**	**39**
**253.9496**	**O_3NOs**	**–1.04**	**10**
255.9414	O_4Os	–1.04	5.7
**264.9545**	**C_2HO_3Os**	**–0.55**	**100**
264.9907	$C_3H_5O_2Os$	–1.14	7.4
**266.9700**	**$C_2H_3O_3Os$**	**–1.07**	**29**
268.9492	CHO_4Os	–1.08	8.6
283.9727	$C_2H_4O_4Os$	–1.08	6.9
**292.9492**	**C_3HO_4Os**	**–0.92**	**12**
**294.9649**	**$C_3H_3O_4Os$**	**–1.02**	**15**
**310.9598**	**$C_3H_3O_5Os$**	**–0.98**	**17**
338.9548	$C_4H_3O_6Os$	–0.68	8.0
354.9497	$C_4H_3O_7Os$	–0.61	7.4
371.9524	$C_4H_4O_8Os$	–0.76	5.1
**382.9445**	**$C_5H_3O_8Os$**	**–0.73**	**22**

[a] Signal intensity of the most abundant ion corresponds to 3.0 × 10^6 counts

Table S13. Ions corresponding to ^{192}Os with their relative intensities detected in the positive ion mode during FI-PVG of Os using 30% (v/v) HCOOH (20Cd) and the confined setup of the DART. Conditions: 1 mg L^{-1} Os taken for PVG, sample flow rate 2 mL min^{-1}; N_2 discharge gas temperature 150 °C, ion transfer tube temperature 200 °C. Ions of relative intensity lower than 5% not shown.

Mass (*m/z*)	Elemental composition	Δ*m/z* (ppm)	Relative intensity[a] (%)
**320.9430**	**C_4HO_5Os**	**–1.12**	**11**
**326.9774**	**$C_3H_5O_5NOs$**	**–0.90**	**14**
337.9459	$C_4H_2O_6Os$	–0.65	6.3
**337.9696**	**$C_4H_4O_5NOs$**	**–0.91**	**40**
**338.9536**	**$C_4H_3O_6Os$**	**–0.92**	**23**
348.9382	C_5HO_6Os	–0.24	7.1
**348.9492**	**$C_4HO_5N_2Os$**	**–0.88**	**12**
355.9564	$C_4H_4O_7Os$	–0.60	5.8
355.9801	$C_4H_6O_6NOs$	–0.91	6.8
**366.9485**	**$C_5H_3O_7Os$**	**–0.79**	**100**

[a] Signal intensity of the most abundant ion corresponds to 4.4×10^6 counts

Table S14. Ions corresponding to ^{192}Os with their relative intensities detected in the negative ion mode during FI-PVG of Os using 30 % (v/v) HCOOH (20Cd) and the confined setup of the DART. Conditions: 1 mg L^{-1} Os taken for PVG, sample flow rate 2 mL min^{-1}; N_2 discharge gas temperature 50 °C, ion transfer tube temperature 200 °C. Ions of relative intensity lower than 5% not shown.

Mass (*m/z*)	Elemental composition	Δ*m/z* (ppm)	Relative intensity[a] (%)
223.9519	O_2Os	0.02	7.5
224.9597	HO_2Os	0.08	9.2
**236.9597**	**CHO_2Os**	**0.16**	**17**
**238.9628**	**HO_2NOs**	**0.14**	**15**
238.9753	CH_3O_2Os	–0.05	8.1
239.9468	O_3Os	0.08	6.4
239.9832	CH_4O_2Os	0.06	5.1
**250.9753**	**$C_2H_3O_2Os$**	**–0.05**	**24**

**251.9468**	**CO_3Os**	**0.23**	**22**
**253.9499**	**O_3NOs**	**0.14**	**16**
263.9469	C_2O_3Os	0.45	5.6
**264.9547**	**C_2HO_3Os**	**0.20**	**79**
265.9501	CO_3NOs	0.77	5.2
**266.9703**	**$C_2H_3O_3Os$**	**0.05**	**17**
268.9495	CHO_4Os	0.07	8.0
280.9496	C_2HO_4Os	0.18	6.0
283.9730	$C_2H_4O_4Os$	0.12	7.8
**292.9495**	**C_3HO_4Os**	**0.10**	**24**
293.9574	$C_3H_2O_4Os$	0.18	5.8
**294.9652**	**$C_3H_3O_4Os$**	**0.23**	**12**
**309.9398**	**C_2O_5NOs**	**0.37**	**17**
**310.9601**	**$C_3H_3O_5Os$**	**0.18**	**18**
311.9555	$C_2H_2O_5NOs$	0.66	8.1
327.9504	$C_2H_2O_6NOs$	0.34	8.2
**338.9552**	**$C_4H_3O_6Os$**	**0.68**	**12**
342.9501	$C_3H_3O_7Os$	0.69	5.8
**352.9347**	**C_4HO_7Os**	**1.12**	**16**
**354.9501**	**$C_4H_3O_7Os$**	**0.61**	**17**
355.9457	$C_3H_2O_7NOs$	1.62	6.2
**371.9405**	**$C_3H_2O_8NOs$**	**1.16**	**13**
**371.9527**	**$C_4H_4O_8Os$**	**0.07**	**14**
**382.9451**	**$C_5H_3O_8Os$**	**0.68**	**100**
**399.9352**	**$C_4H_2O_9NOs$**	**0.57**	**35**
400.9558	$C_5H_5O_9Os$	1.01	8.3

[a] Signal intensity of the most abundant ion corresponds to 7.1×10^6 counts

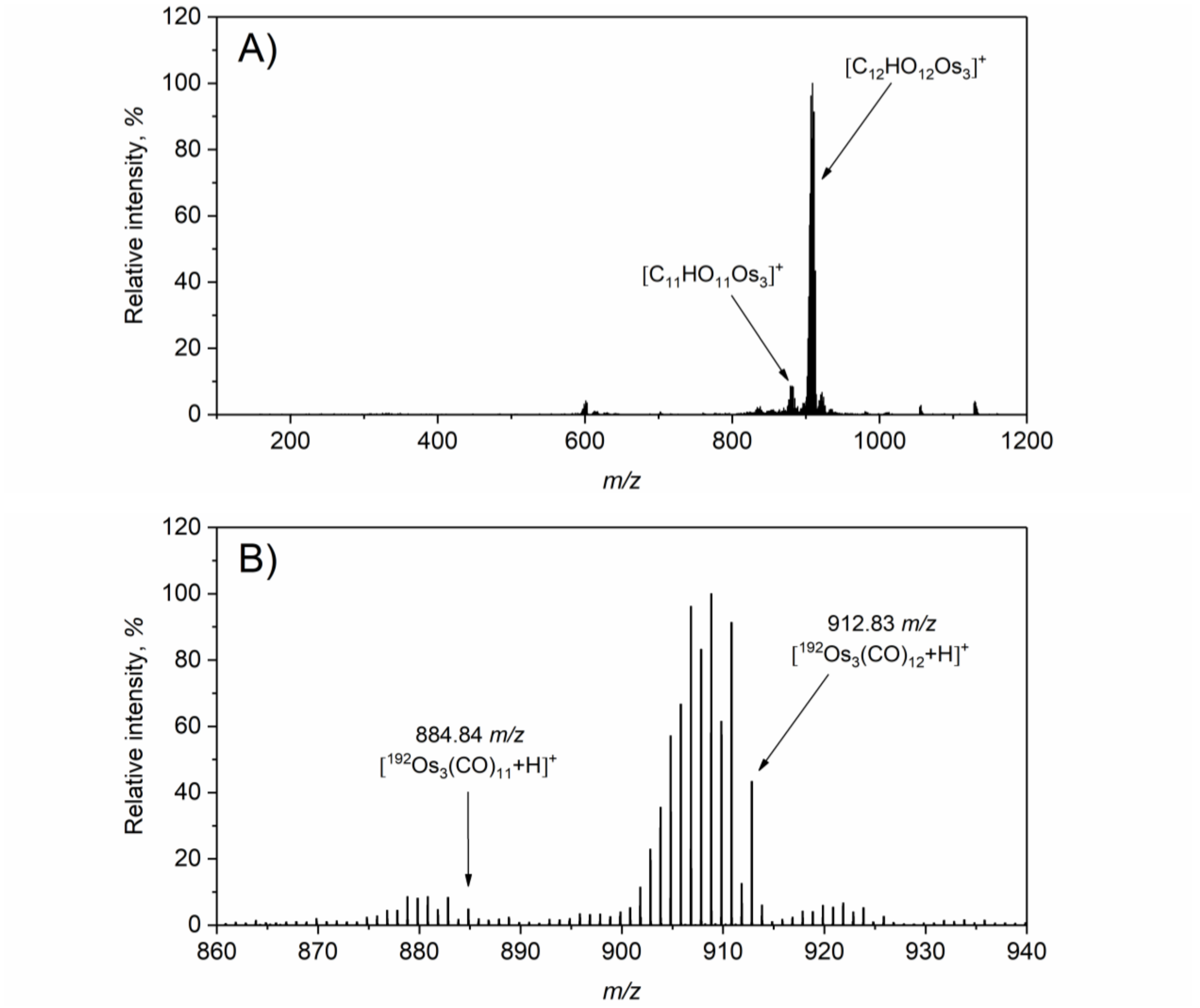

Figure S6. A) Full scan DART-HRMS spectrum obtained in the positive ion mode from the headspace sampling of preheated (125 °C) solid $Os_3(CO)_{12}$ introduced directly to the DART and B) a close-up of the isotopic patterns of two major ions (860–940 *m/z*) with specified ions containing only ^{192}Os. Conditions: PVG operated with 30% (v/v) HCOOH, sample flow rate 2 mL min^{-1}; N_2 discharge gas temperature 350 °C, ion transfer tube temperature 300 °C.

Oxidative conditions for PVG of Os. Mainly three major ions were detected during PVG from 5% (v/v) HNO_3, DIW, and 1% (v/v) H_2O_2),[8–10] namely $[O_3Os]^-$, $[O_3NOs]^-$, and $[O_4Os]^-$, with some differences in their relative abundances (Figure S7A,B,C and Table S15, S16, and S17). $[O_4Os]^-$ was the dominant ion in the spectrum during PVG from 5% (v/v) HNO_3, while the relative abundances of all the three major ions were above 50% for the other two media. This difference is not entirely clear but may be due to the different "acidity" of the gas phase introduced into the DART, since some aerosol (droplets) of the liquid photochemical media is produced in the GLS I and transported into the DART by Ar carrier. This is also indicated by the presence of $[HO_6NOs]^-$ detected under PVG from 5% (v/v) HNO_3, possibly due to formation of NO_3^- adduct. Evidence that $[O_3NOs]^-$ is indeed produced in the DART due to the use of N_2 as the discharge gas was provided by an experiment when PVG was performed from DIW and DART was operated with Ar as the discharge gas. Only $[O_4Os]^-$ was detected but the measurements with Ar as the discharge gas were characterized by ≈200 times lower sensitivity. It was reported that the poor sensitivity of the Ar DART can be substantially enhanced when some organic dopant (methanol, acetone, toluene, etc.) is introduced into the DART stream.[11,12] A means of effectively introducing an organic dopant was not properly investigated in this work but when the vial of methanol was placed directly under the Ar stream exiting the DART source, the sensitivity increased 10-fold compared to the pure Ar. In this case, the most abundant ion was still $[O_4Os]^-$ and also $[O_3Os]^-$ could be detected (1.5% relative intensity), but absolutely no $[O_3NOs]^-$. This provides evidence that $[O_3NOs]^-$ is specifically formed from OsO_4 in the N_2 DART.

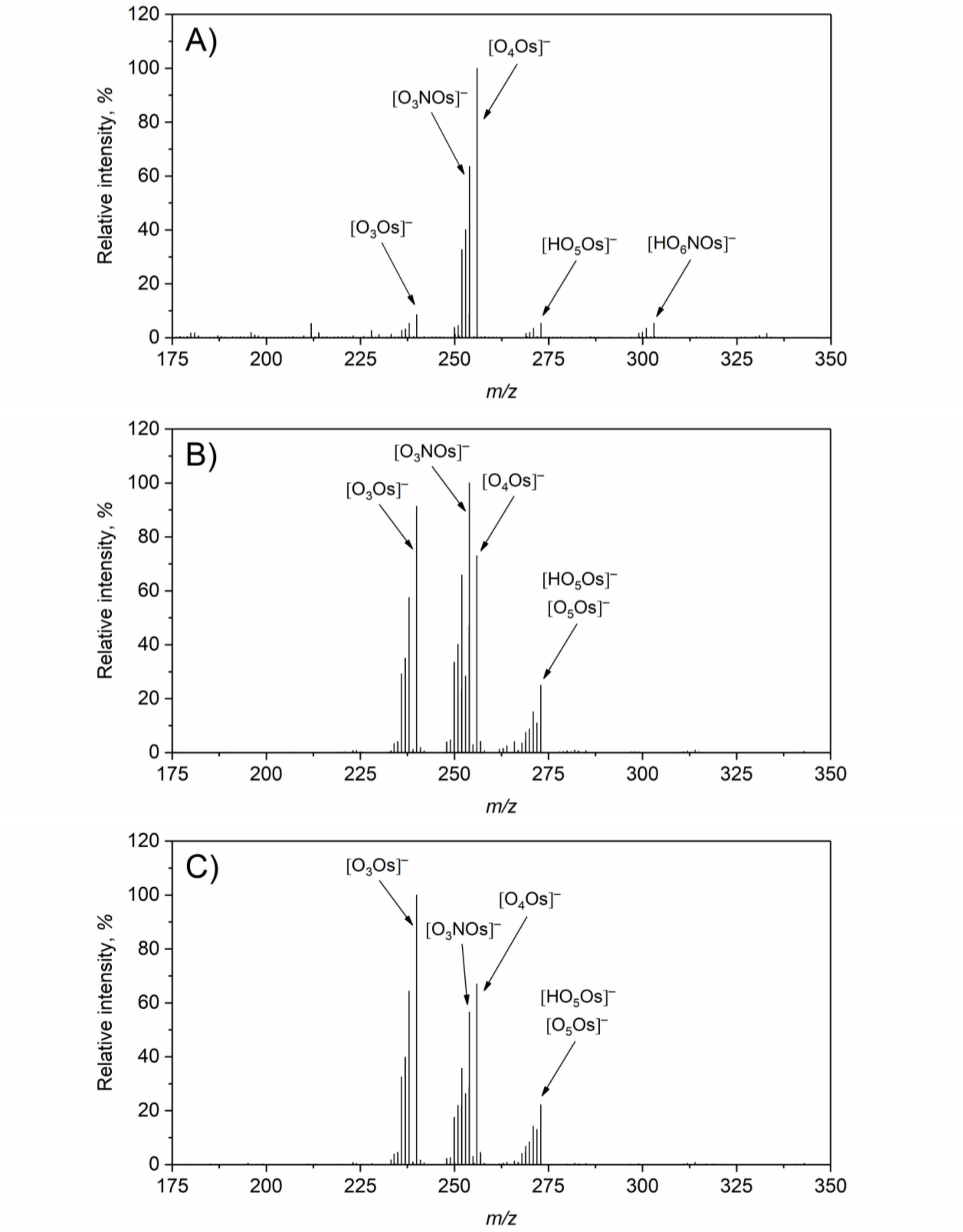

Figure S7. Full scan DART-HRMS spectra obtained in the negative ion mode using the open setup of the DART during FI-PVG of Os using A) 5% (v/v) HNO_3, B) DIW, and C) 1% (v/v) H_2O_2. Conditions: 1 mg L^{-1} Os taken for PVG, sample flow rate 2 mL min^{-1}; N_2 discharge gas temperature 150 °C, ion transfer tube temperature 300 °C. See Tables S15, S16, and S17 for exact values of relative abundances.

Table S15. Ions corresponding to ^{192}Os with their relative intensities detected in the negative ion mode during FI-PVG of Os using 5% (v/v) HNO_3 and the open setup of the DART. Conditions: 1 mg L^{-1} Os taken for PVG, sample flow rate 2 mL min^{-1}; N_2 discharge gas temperature 150 °C, ion transfer tube temperature 300 °C. Ions of relative intensity lower than 5% not shown.

Mass (m/z)	Elemental composition	$\Delta m/z$ (ppm)	Relative intensity (%)
239.9467	O_3Os	–0.51	8.5
**253.9497**	**O_3NOs**	**–0.45**	**11**
**255.9416**	**O_4Os**	**–0.34**	**100**
272.9443	HO_5Os	–0.53	5.3
302.9423	HO_6NOs	–0.34	5.3

[a] Signal intensity of the most abundant ion corresponds to 5.5×10^5 counts

Table S16. Ions corresponding to ^{192}Os with their relative intensities detected in the negative ion mode during FI-PVG of Os using DIW and the open setup of the DART. Conditions: 1 mg L^{-1} Os taken for PVG, sample flow rate 2 mL min^{-1}; N_2 discharge gas temperature 150 °C, ion transfer tube temperature 300 °C. Ions of relative intensity lower than 5% not shown.

Mass (m/z)	Elemental composition	$\Delta m/z$ (ppm)	Relative intensity (%)
**239.9467**	**O_3Os**	**–0.17**	**91**
**253.9498**	**O_3NOs**	**–0.26**	**100**
**255.9415**	**O_4Os**	**–0.57**	**73**
**271.9364**	**O_5Os**	**–0.81**	**11**
**272.9442**	**HO_5Os**	**–0.79**	**25**

[a] Signal intensity of the most abundant ion corresponds to 2.7×10^6 counts

Table S17. Ions corresponding to ^{192}Os with their relative intensities detected in the negative ion mode during FI-PVG of Os using 1% (v/v) H_2O_2 and the open setup of the DART. Conditions: 1 mg L^{-1} Os taken for PVG, sample flow rate 2 mL min^{-1}; N_2 discharge gas temperature 150 °C, ion transfer tube temperature 300 °C. Ions of relative intensity lower than 5% not shown.

Mass (*m/z*)	Elemental composition	Δ*m/z* (ppm)	Relative intensity[a] (%)
**239.9465**	**O_3Os**	**–1.09**	**100**
**253.9495**	**O_3NOs**	**–1.32**	**57**
**255.9414**	**O_4Os**	**–1.20**	**67**
**271.9362**	**O_5Os**	**–1.58**	**13**
**272.9440**	**HO_5Os**	**–1.45**	**22**

[a] Signal intensity of the most abundant ion corresponds to 1.8×10^6 counts

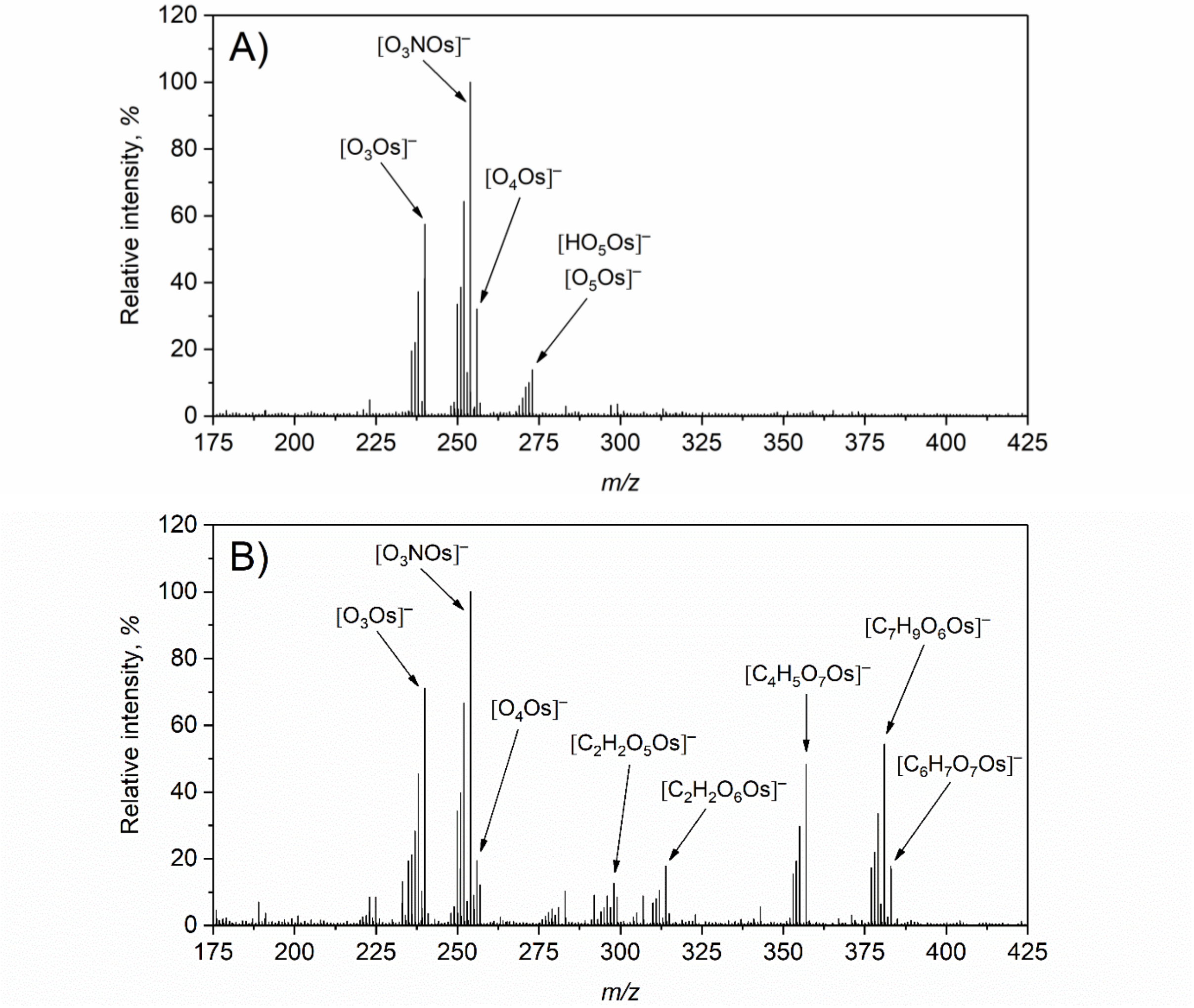

Figure S8. Full scan DART-HRMS spectra obtained in the negative ion mode A) at the beginning and B) after 60 min of FI-PVG of Os using 1% (v/v) CH_3COOH (50Fe) and the open setup of the DART. Conditions: 1 mg L^{-1} Os taken for PVG, sample flow rate 2 mL min^{-1}; N_2 discharge gas temperature 150 °C, ion transfer tube temperature 300 °C. See Tables S18 and S19 for exact values of relative abundances.

Table S18. Ions corresponding to ^{192}Os with their relative intensities detected in the negative ion mode at the beginning of FI-PVG of Os using 1% (v/v) CH_3COOH (50Fe) and the open setup of the DART. Conditions: 1 mg L^{-1} Os taken for PVG, sample flow rate 2 mL min^{-1}; N_2 discharge gas temperature 150 °C, ion transfer tube temperature 300 °C. Ions of relative intensity lower than 5% not shown.

Mass (*m/z*)	Elemental composition	Δ*m/z* (ppm)	Relative intensity[a] (%)
**239.9471**	**O_3Os**	**1.33**	**57**
**253.9502**	**O_3NOs**	**1.44**	**100**
**255.9421**	**O_4Os**	**1.50**	**32**
**271.9371**	**O_5Os**	**1.65**	**10**
**272.9448**	**HO_5Os**	**1.52**	**14**

[a] Signal intensity of the most abundant ion corresponds to 1.0×10^5 counts

Table S19. Ions corresponding to ^{192}Os with their relative intensities detected in the negative ion mode after 60 min of FI-PVG of Os using 1% (v/v) CH_3COOH (50Fe) and the open setup of the DART. Conditions: 1 mg L^{-1} Os taken for PVG, sample flow rate 2 mL min^{-1}; N_2 discharge gas temperature 150 °C, ion transfer tube temperature 300 °C. Ions of relative intensity lower than 5% not shown.

Mass (*m/z*)	Elemental composition	Δ*m/z* (ppm)	Relative intensity[a] (%)
224.9600	HO_2Os	1.55	8.5
238.9757	CH_3O_2Os	1.37	9.9
**239.9471**	**O_3Os**	**1.37**	**71**
**253.9502**	**O_3NOs**	**1.52**	**100**
**255.9421**	**O_4Os**	**1.66**	**19**
**256.9499**	**HO_4Os**	**1.52**	**12**
**282.9656**	**$C_2H_3O_4Os$**	**1.62**	**10**
**297.9528**	**$C_2H_2O_5Os$**	**1.84**	**13**
298.9606	$C_2H_3O_5Os$	1.69	8.4
306.9657	$C_4H_3O_4Os$	1.89	8.8
**313.9477**	**$C_2H_2O_6Os$**	**1.64**	**18**
342.9500	$C_3H_3O_7Os$	0.31	5.4
**356.9657**	**$C_4H_5O_7Os$**	**0.38**	**48**
**381.0023**	**$C_7H_9O_6Os$**	**0.84**	**54**
**382.9815**	**$C_6H_7O_7Os$**	**0.72**	**18**

[a] Signal intensity of the most abundant ion corresponds to 1.1×10^5 counts

Discussion. The ions produced in the DART source during introduction of volatile species of Ru and Os generated under reductive PVG conditions (HCOOH-based photochemical media) appear unstable as they are rapidly attacked by H_2O and O_2 from the ambient atmosphere and the reactive species derived from the use of N_2 as the discharge gas. This appears to be characteristic of the volatile metal carbonyls as such a similar hydration and oxidation of ions has been identified for $W(CO)_6$ and $Fe(CO)_5$, either photochemically generated or introduced as standards in a headspace. Nevertheless, the results indicate that the volatile species of Ru and Os are mononuclear, contain maximum 5 CO groups, and are most likely $Ru(CO)_5$ and $Os(CO)_5$.[13,14] The hypothetical possibility of the coexistence of some hydridocarbonyl species cannot be easily excluded from the DART-HRMS data. This might be true for Os, as it has been reported that $Os(CO)_5$ can be converted to $Os(CO)_4H_2$ in the presence of traces of H_2, which is even more volatile than $Os(CO)_5$.[13,15] Hydrogen is produced in sufficient quantities during UV photolysis of HCOOH media.[16]

The DART-HRMS spectra obtained for the photochemically generated volatile species of Ru and Os are significantly different from those arising from $Ru_3(CO)_{12}$ and $Os_3(CO)_{12}$ introduced directly into the DART and measured under identical conditions (with concurrent PVG operation). This result is contradictory to the paper by Yang et al.[17] who tentatively identified volatile species of Ru using ESI-HRMS after their cryogenic trapping in acetonitrile and derivatization with Ag^+ according to a previously reported protocol.[18,19] Their ESI-HRMS spectra indicated the presence of polynuclear Ru species, most likely $Ru_3(CO)_{12}$, but the results were not entirely convincing according to the authors, hence, they only hypothesized the presence of some $Ru_x(CO)_y$ species.[17] As mentioned in the Introduction, the weakness of this derivatization approach based on Ag^+ addition is that it is only suitable for ESI ionization of polynuclear metal carbonyls and not of simple mononuclear carbonyls such as $W(CO)_6$.[18,19] Due to the instability of $Ru(CO)_5$, there is a serious risk of clustering to $Ru_3(CO)_{12}$ after trapping it in the liquid acetonitrile. Our results are supported by the experiments in which it was demonstrated that $Ru_3(CO)_{12}$ and $Os_3(CO)_{12}$ do not have sufficient volatility,[20] and cannot be transported from the generator in the gas phase.

The same ions derived from volatile species of Ru were detected during PVG from both dilute HCOOH and HCOONa media, as well as from concentrated HCOOH in the presence of Cd^{2+} as a mediator, still indicating $Ru(CO)_5$ as the volatile species, which rules out a significant effect of HCOOH concentration on the measured DART-HRMS spectra. Finally, the major ions were also detected under "analytical conditions" using high concentrations of HCOOH in the presence of Co^{2+} and Cd^{2+} as mediators, although co-generation of $Co(CO)_4H$ and overloading of the DART-HRMS made identification difficult. In addition to many Co-containing ions, several Ru-Co-

containing ions were also detected (see the "Ruthenium" section of the main article). The tantalizing question is whether the formation of such mixed metal carbonyl clusters cannot occur during PVG. Indeed, it could explain a significant enhancement in PVG due to the presence of Co^{2+} and the formation of a new mixed metal carbonyl species, with more favorable physical and chemical properties than the original Ru and Os pentacarbonyls. An additional experiment was therefore devoted to clarifying this phenomenon for Ru as the analyte. Two almost identical PVG systems were used in parallel and the gas phases exiting each GLS I were mixed in a T-piece upstream of the GLS II (only one GLS II used). $Ru(CO)_5$ was generated separately in the first generator by FI-PVG using 0.01 M HCOOH (10Cd), while $Co(CO)_4H$ was generated in the second system using 8 M HCOOH (10Co/25Cd) and a continuous flow mode of operation. Exactly the same Ru-Co ions, as listed in the "Ruthenium" section of the main article, and several $Ru\text{-}Co_{2\text{-}3}$ ions were detected with this parallel setup. It is clear that the clustering does not occur during PVG but during ionization of $Ru(CO)_5$ and $Co(CO)_4H$ in the DART.

Potentially controversial results were encountered for identification of volatile species of Os during PVG from 1% (v/v) CH_3COOH (50Fe), as reported by de Oliveira et al.[8] These authors concluded that such a medium would give rise to reductive conditions, possibly producing a hypothetical volatile species $Os_w(CO)_x(CH_3)_yH_z$, with physical properties different from OsO_4. Unfortunately, osmium species could not be identified by GC-MS experiments in a follow-up study.[21] To the contrary, DART-HRMS data indicate rather oxidative PVG conditions for this medium, with production of OsO_4 as the dominant volatile species. Our evidence for OsO_4 is in a full agreement with the work by Yang et al.[17] who also demonstrated the generation of OsO_4 from this medium and DIW by oxidation of quercetin to quinone species by generated OsO_4. Differences in the purity of the stock CH_3COOH used in different laboratories, especially the presence of traces of HCOOH, might be the reason for this discrepancy. The highly efficient PVG of Ru, Re, and Ir was recently demonstrated from very dilute HCOOH medium (0.01 M),[1] and the traces of HCOOH could cause the PVG conditions to change from oxidative to reductive. Additional experiments with ICPMS as the detector ($^{192}Os^+$ and $^{189}Os^+$ monitored) were therefore focused on the effect of added HCOOH in the range of 0.0001–0.01 M to 1% (v/v) CH_3COOH (50Fe). No indication of any change to reductive PVG conditions was evident, as no significant improvement in the PVG yield in the presence of the added 0.0001 M HCOOH was noted; rather, the effects of 0.001 M and 0.01 M HCOOH were strongly negative.

Additional insight was gained by comparing the measured transient signal profiles (FI peaks) using ICPMS as the detector following PVG of Os from 5% (v/v) HNO_3, 1% (v/v) CH_3COOH (50Fe), and 30% (v/v) HCOOH (10Co/20Cd) (see Figure S9).

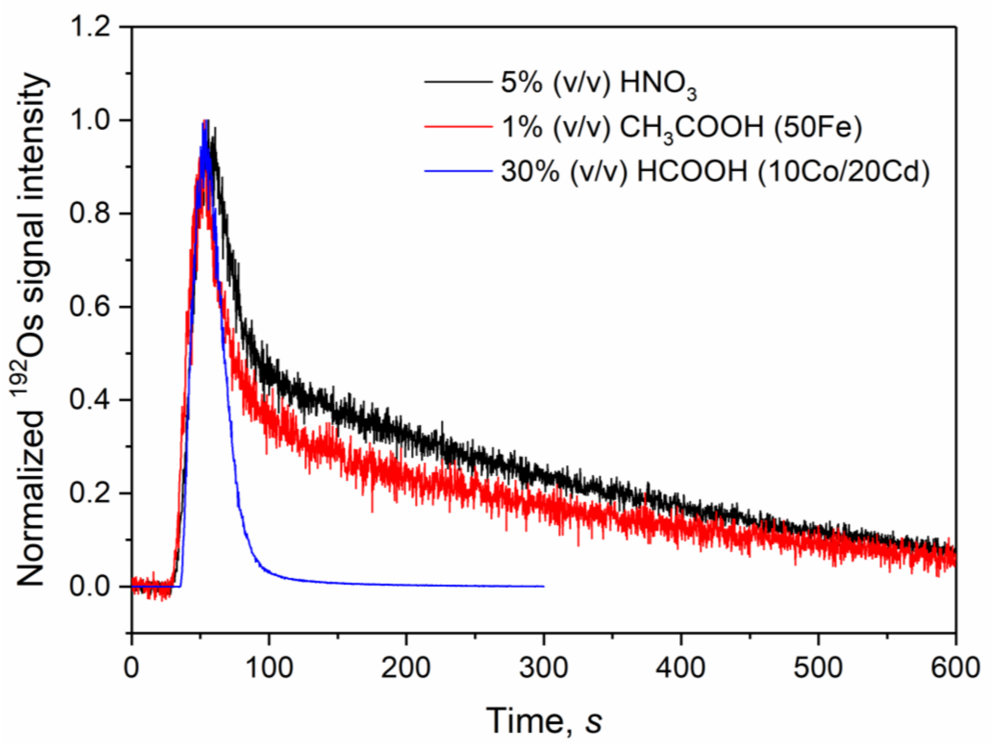

Figure S9. Comparison of normalized transient ICPMS signals obtained during PVG of Os using 5% (v/v) HNO_3, 1% (v/v) CH_3COOH (50Fe), and 30% (v/v) HCOOH (10Co/20Cd). Conditions: 0.5 µg L^{-1} Os taken for PVG, sample flow rate 2 mL min^{-1}.

The transient signal profiles for 5% (v/v) HNO_3 and 1% (v/v) CH_3COOH (50Fe) were characterized by an extensive tailing, taking about 10 min to reach the baseline completely after sample injection. The long washout times have been already described for PVG of Os using oxidative conditions and resulting in OsO_4 as the volatile species in the earlier reports.[8–10] It was found in this work that the washout period could be shortened to about 5 min when the GLS I was not immersed in the ice-bath. However, the FI peaks measured with 30% (v/v) HCOOH (10Co/20Cd), i.e., under strictly reductive conditions, were much sharper and the decline to baseline took no more than 3 min. Absolutely the same transient signal characteristics for reductive and oxidative conditions were evident for individual ion intensities measured at defined *m/z* using DART-HRMS, with extensive tailing for all ions listed in Tables S15, S16, S17, and S18. The same tailing characteristics were evident for the majority of C-containing ions detected after prolonged PVG with 1% (v/v) CH_3COOH (Table S19), namely $[CH_3O_2Os]^-$, $[C_2H_3O_4Os]^-$, $[C_2H_2O_5Os]^-$, $[C_2H_2O_6Os]^-$, and $[C_4H_5O_7Os]^-$. These ions may originate in the DART or during transport, upon mixing generated OsO_4 with reducing components of the gas phase derived from the UV photolysis of CH_3COOH (CO, CO_2, CH_4, and C_2H_6).[16] Conversely, the signal intensities for $[C_7H_9O_6Os]^-$ and $[C_6H_7O_7Os]^-$ returned to baseline as quickly as for ions detected under

reductive conditions. In order to determine whether some of the ions were formed in the DART or were derived from other volatile species generated by PVG, the measurement was again performed with two PVG systems in parallel. OsO_4 was generated separately in the first FI-PVG system using DIW as the photochemical medium while 1% (v/v) CH_3COOH was continuously delivered to the second PVG system and the resulting gas phases were mixed in the T-piece upstream of the GLS II. Neither $[C_7H_9O_6Os]^-$ nor $[C_6H_7O_7Os]^-$ was detected in the spectra using this setup, in contrast to $[C_2H_3O_4Os]^-$, $[C_2H_2O_5Os]^-$, $[C_2H_2O_6Os]^-$, and $[C_4H_5O_7Os]^-$, which were readily observed and for which the tailing characteristics similar to those of OsO_4 were recorded. This is further evidence that some other volatile species are generated.

MS^2 experiments with $[C_7H_9O_6Os]^-$ at 381.00 *m/z* as the precursor ion and using 20% normalized collision energy lead mainly to $[C_5H_5O_4Os]^-$ (100%) at 320.98 *m/z* as the fragment ion, but also to $[C_6H_9O_4Os]^-$ (17%) at 337.01 *m/z*, $[C_5H_3O_4Os]^-$ (16%) at 318.97 *m/z*, and $[C_4H_5O_3Os]^-$ (36%) at 292.91 *m/z*. Their interpretation for structure identification is not straightforward because the precursor ion is most likely an adduct formed in the DART, as demonstrated above for $Ru(CO)_5$ and $Os(CO)_5$. For example, $[C_7H_9O_6Os]^-$ could be an OH^- adduct, as suggested by a loss of CO_2 due to gas-shift reaction[22] and the appearance of $[C_6H_9O_4Os]^-$. Another possibility could be a CH_3COO^- adduct due to the presence of CH_3COOH vapor in the gas phase, as indicated by the presence of the dominant $[C_5H_5O_4Os]^-$ and $[C_2H_3O_2]^-$ (48%) at 59.01 *m/z*. The loss of H_2 is evident from $[C_5H_5O_4Os]^-$ and $[C_5H_3O_4Os]^-$, while the loss of CO group is evident from $[C_5H_5O_4Os]^-$ and $[C_4H_5O_3Os]^-$. In addition, the loss of the CH_3 group can be speculated based on the detected $[C_6H_9O_4Os]^-$ and $[C_5H_5O_4Os]^-$, for which the difference in their formulas actually corresponds to CH_4. However, a CH_3 group bonded to the central Os atom requires a bonded hydride ligand.[23] Similar MS^2 experiments using $[C_6H_7O_7Os]^-$ at 382.98 *m/z* as the precursor ion showed a similar loss of CH_3COOH by the appearance of $[C_4H_3O_5Os]^-$ (100%) at 322.96 *m/z*. In summary, it appears that the volatile species contains at least one CO group, and possibly also H_2 (similar as in the case of $Os(CO)_4H_2$), but it is speculative as to whether it contains a bound acetate ligand[24] or a methyl group,[23] both of which would require an additional hydride ligand.

The full structure of this compound is thus not completely clear and identification could not be further advanced due to insufficient PVG efficiency and resulting low ion intensities. It is also not clear whether $[C_6H_7O_7Os]^-$ and $[C_7H_9O_6Os]^-$ can be attributed to a single volatile species or whether they reflect the presence of two different volatile species generated during PVG (for example, $Os(CO)_4(CH_3)_2$ and $Os(CO)_4(CH_3)H$).[23] In any case, it is curious why the volatile species cannot be detected in the positive ion mode and why its formation is initiated after prolonged PVG with dilute CH_3COOH.

REFERENCES

(1) Jeníková, E.; Vyhnanovský, J.; Hašlová, K.; Sturgeon, R. E.; Musil, S. Efficient Photochemical Vapor Generation from Low Concentration Formic Acid Media. *Anal. Chem.* **2024**, *96*, 1241–1250.

(2) Musil, S.; Jeníková, E.; Vyhnanovský, J.; Sturgeon, R. E. Highly Efficient Photochemical Vapor Generation for Sensitive Determination of Iridium by Inductively Coupled Plasma Mass Spectrometry. *Anal. Chem.* **2023**, *95*, 3694–3702.

(3) Musil, S.; Vyhnanovský, J.; Sturgeon, R. E. Ultrasensitive Detection of Ruthenium by Coupling Cobalt and Cadmium Ion-Assisted Photochemical Vapor Generation to Inductively Coupled Plasma Mass Spectrometry. *Anal. Chem.* **2021**, *93*, 16543–16551.

(4) Vyhnanovský, J.; Sturgeon, R. E.; Musil, S. Cadmium Assisted Photochemical Vapor Generation of Tungsten for ICPMS detection. *Anal. Chem.* **2019**, *91*, 13306–13312.

(5) Nemykin, V. N.; Basu, P. Energy-dependent electrospray ionization mass spectrometric studies of mononuclear metal carbonyls. *Inorg. Chim. Acta* **2005**, *358*, 2876–2882.

(6) Yang, C.; Yao, S.; Zheng, H.; Zhu, Z. Determination of Trace Fe in Water Sample by Atmospheric Pressure Glow Discharge Microplasma Coupled with Photochemical Vapor Generation. *Spectrosc. Spect. Anal.* **2019**, *39*, 1366–1371.

(7) Gross, J. H. Direct analysis in real time-a critical review on DART-MS. *Anal. Bioanal. Chem.* **2014**, *406*, 63–80.

(8) de Oliveira, R. M.; Borges, D. L. G.; Grinberg, P.; Sturgeon, R. E. High-efficiency photoreductive vapor generation of osmium. *J. Anal. At. Spectrom.* **2021**, *36*, 2097–2106.

(9) Gao, Y.; Li, S.; He, H.; Li, T.; Yu, T.; Liu, R.; Ni, S.; Shi, Z. Sensitive determination of osmium in natural waters by inductively coupled plasma mass spectrometry after photochemical vapor generation. *Microchem. J.* **2017**, *130*, 281–286.

(10) Zhu, Z.; He, D.; Huang, C.; Zheng, H.; Zhang, S.; Hu, S. High-efficiency photooxidation vapor generation of osmium for determination by inductively coupled plasma-optical emission spectrometry. *J. Anal. At. Spectrom.* **2014**, *29*, 506–511.

(11) Yang, H.; Wan, D.; Song, F.; Liu, Z.; Liu, S. Argon Direct Analysis in Real Time Mass Spectrometry in Conjunction with Makeup Solvents: A Method for Analysis of Labile Compounds. *Anal. Chem.* **2013**, *85*, 1305–1309.

(12) Cody, R. B.; Dane, A. J. Dopant-assisted direct analysis in real time mass spectrometry with argon gas. *Rapid Commun. Mass Spectrom.* **2016**, *30*, 1181–1189.

(13) Hieber, W.; Stallmann, H. Über Metallcarbonyle. XLVI. Über Osmiumcarbonyle. *Z. Elektrochem. Angew. Phys. Chem.* **1943**, *49*, 288–292.

(14) Manchot, W.; Manchot, W. J. Darstellung von Rutheniumcarbonylen und -nitrosylen. *Z. Anorg. Allg. Chem.* **1936**, *226*, 385–415.

(15) L'Eplattenier, F.; Calderazzo, F. Pentacarbonyls of ruthenium and osmium. II. Dihydridotetracarbonylosmium and its substitution reactions. *Inorg. Chem.* **1967**, *6*, 2092–2097.

(16) Sturgeon, R. E.; Pagliano, E.; Lopes, G. S.; Neto, R. S. A.; Brito, J. K. S. Insights into the role of transition and noble metals mediating photochemical vapor generation. *J. Anal. At. Spectrom.* **2025**, *40*, 70–97.

(17) Yang, Q.; Chen, H.; Hu, J.; Huang, K.; Hou, X. Simultaneous Detection of Ruthenium and Osmium by Photochemical Vapor Generation-Inductively Coupled Plasma-Mass Spectrometry. *Anal. Chem.* **2022**, *94*, 593–599.

(18) Henderson, W.; Nicholson, B. K. Electrospray mass spectrometry of neutral metal carbonyl complexes using silver(I) ions for ionisation. *J. Chem. Soc., Chem. Commun.* **1995**, 2531–2532.

(19) Henderson, W.; Scott McIndoe, J.; K. Nicholson, B.; J. Dyson, P. Electrospray mass spectrometry of metal carbonyl complexes†. *J. Chem. Soc., Dalton Trans.* **1998**, 519–526.

(20) Fillman, L. M.; Tang, S. C. Thermal decomposition of metal carbonyls: A thermogravimetry-mass spectrometry study. *Thermochim. Acta* **1984**, *75*, 71–84.

(21) Pagliano, E.; Vyhnanovský, J.; Musil, S.; de Oliveira, R. M.; Forczek, S. T.; Sturgeon, R. E. GC-MS exploration of photochemically generated species of Os, W and Ru from reductive and oxidative media. *J. Anal. At. Spectrom.* **2022**, *37*, 528–534.

(22) Rozanska, X.; Vuilleumier, R. Mechanisms of the Water−Gas-Shift Reaction by Iron Pentacarbonyl in the Gas Phase. *Inorg. Chem.* **2008**, *47*, 8635–8640.

(23) L'Eplattenier, F. Pentacarbonyls of ruthenium and osmium. IV. Synthesis of alkyl- and acetylosmium carbonyls. *Inorg. Chem.* **1969**, *8*, 965–970.

(24) Robinson, S. D.; Uttley, M. F. Complexes of the platinum metals. Part II. Carboxylato(triphenylphosphine) derivatives of ruthenium, osmium, rhodium, and iridium. *J. Chem. Soc., Dalton Trans.* **1973**, 1912–1920.
